# Pathways-Driven Sparse Regression Identifies Pathways and Genes Associated with High-Density Lipoprotein Cholesterol in Two Asian Cohorts

**DOI:** 10.1371/journal.pgen.1003939

**Published:** 2013-11-21

**Authors:** Matt Silver, Peng Chen, Ruoying Li, Ching-Yu Cheng, Tien-Yin Wong, E-Shyong Tai, Yik-Ying Teo, Giovanni Montana

**Affiliations:** 1 Statistics Section, Department of Mathematics, Imperial College, London, United Kingdom; 2 MRC International Nutrition Group, London School of Hygiene and Tropical Medicine, London, United Kingdom; 3 Saw Swee Hock School of Public Health, National University of Singapore, Singapore; 4 Yong Loo Lin School of Medicine, National University of Singapore, Singapore; 5 Department of Ophthalmology, National University of Singapore, Singapore; 6 Singapore Eye Research Institute, Singapore National Eye Center, Singapore; 7 NUS Graduate School for Integrative Science and Engineering, National University of Singapore, Singapore; 8 Life Sciences Institute, National University of Singapore, Singapore; 9 Genome Institute of Singapore, Agency for Science, Technology and Research, Singapore; 10 Department of Statistics and Applied Probability, National University of Singapore, Singapore; Dartmouth College, United States of America

## Abstract

Standard approaches to data analysis in genome-wide association studies (GWAS) ignore any potential functional relationships between gene variants. In contrast gene pathways analysis uses prior information on functional structure within the genome to identify pathways associated with a trait of interest. In a second step, important single nucleotide polymorphisms (SNPs) or genes may be identified within associated pathways. The pathways approach is motivated by the fact that genes do not act alone, but instead have effects that are likely to be mediated through their interaction in gene pathways. Where this is the case, pathways approaches may reveal aspects of a trait's genetic architecture that would otherwise be missed when considering SNPs in isolation. Most pathways methods begin by testing SNPs one at a time, and so fail to capitalise on the potential advantages inherent in a multi-SNP, joint modelling approach. Here, we describe a dual-level, sparse regression model for the simultaneous identification of pathways and genes associated with a quantitative trait. Our method takes account of various factors specific to the joint modelling of pathways with genome-wide data, including widespread correlation between genetic predictors, and the fact that variants may overlap multiple pathways. We use a resampling strategy that exploits finite sample variability to provide robust rankings for pathways and genes. We test our method through simulation, and use it to perform pathways-driven gene selection in a search for pathways and genes associated with variation in serum high-density lipoprotein cholesterol levels in two separate GWAS cohorts of Asian adults. By comparing results from both cohorts we identify a number of candidate pathways including those associated with cardiomyopathy, and T cell receptor and PPAR signalling. Highlighted genes include those associated with the L-type calcium channel, adenylate cyclase, integrin, laminin, MAPK signalling and immune function.

## Author Summary

Genes do not act in isolation, but interact in complex networks or pathways. By accounting for such interactions, pathways analysis methods hope to identify aspects of a disease or trait's genetic architecture that might be missed using more conventional approaches. Most existing pathways methods take a univariate approach, in which each variant within a pathway is separately tested for association with the phenotype of interest. These statistics are then combined to assess pathway significance. As a second step, further analysis can reveal important genetic variants within significant pathways. We have previously shown that a joint-modelling approach using a sparse regression model can increase the power to detect pathways influencing a quantitative trait. Here we extend this approach, and describe a method that is able to simultaneously identify pathways and genes that may be driving pathway selection. We test our method using simulations, and apply it to a study searching for pathways and genes associated with high-density lipoprotein cholesterol in two separate East Asian cohorts.

## Introduction

Much attention continues to be focused on the problem of identifying SNPs and genes influencing a quantitative or dichotomous trait in genome wide scans [1]. Despite this, in many instances gene variants identified in GWAS have so far uncovered only a relatively small part of the known heritability of most common diseases [2]. Possible explanations include the presence of multiple SNPs with small effects, or of rare variants, which may be hard to detect using conventional approaches [2]–[4].

One potentially powerful approach to uncovering the genetic etiology of disease is motivated by the observation that in many cases disease states are likely to be driven by multiple genetic variants of small to moderate effect, mediated through their interaction in molecular networks or pathways, rather than by the effects of a few, highly penetrant mutations [5]. Where this assumption holds, the hope is that by considering the joint effects of variants acting in concert, pathways GWAS methods will reveal aspects of a disease's genetic architecture that would otherwise be missed when considering variants individually [6], [7]. In this paper we describe a sparse regression method utilising prior information on gene pathways to identify putative causal pathways, along with the constituent variants that may be driving pathways association.

Sparse modelling approaches are becoming increasingly popular for the analysis of genome wide datasets [8]–[11]. Sparse regression models enable the joint modelling of large numbers of SNP predictors, and perform ‘model selection’ by highlighting small numbers of variants influencing the trait of interest. These models work by penalising or constraining the size of estimated regression coefficients. An interesting feature of these methods is that different sparsity patterns, that is different sets of genetic predictors having specified properties, can be obtained by varying the nature of this constraint. For example, the lasso [12] selects a subset of variants whose main effects best predict the response. Where predictors are highly correlated, the lasso tends to select one of a group of correlated predictors at random. In contrast, the elastic net [13] selects groups of correlated variables. Model selection may also be driven by external information, unrelated to any statistical properties of the data being analysed. For example, the fused lasso [14], [15] uses ordering information, such as the position of genomic features along a chromosome to select ‘adjacent’ features together.

Prior information on functional relationships between genetic predictors can also be used to drive the selection of groups of variables. In the present context, information mapping genes and SNPs to functional gene pathways has recently been used in sparse regression models for pathway selection. Chen et al. [16] describe a method that uses a combination of lasso and ridge regression to assess the significance of association between a candidate pathway and a dichotomous (case-control) phenotype, and apply this method in a study of colon cancer etiology. In contrast, Silver et al. [17] use group lasso penalised regression to select pathways associated with a multivariate, quantitative phenotype characteristic of structural change in the brains of patients with Alzheimer's disease.

In identifying pathways associated with a trait of interest, a natural follow-up question is to ask which SNPs and/or genes are driving pathway selection? We might further ask a related question: can the use of prior information on putative gene interactions within pathways increase power to identify causal SNPs or genes, compared to alternative methods that disregard such information? One way to answer these questions is by conducting a two-stage analysis, in which we first identify important pathways, and then in a second step search for SNPs or genes within selected pathways [18], [19]. There are however a number of problems with this approach. Firstly, highlighted variants are then not necessarily those that were driving pathway selection in the first step of the analysis. Secondly, the implicit (and reasonable) assumption is that only a small number of SNPs in a pathway are driving pathway selection, so that ideally we would prefer a model that has this assumption built in. The above considerations point to the use of a ‘dual-level’ sparse regression model that imposes sparsity at both the pathway and SNP level. Such a model would perform *simultaneous* pathway and SNP selection, with the additional benefit of being simpler to implement.

A suitable sparse regression model enforcing the required dual-level sparsity is the sparse group lasso (SGL) [20]. SGL is a comparatively recent development in sparse modelling, and in simulations has been shown to accurately recover dual-level sparsity, in comparison to both the group lasso and lasso [20], [21]. SGL has been used for the identification of rare variants in a case-control study by grouping SNPs into genes [22]; for the identification of genomic regions whose copy number variations have an impact on RNA expression levels [23]; and to model geographical factors driving climate change [24]. SGL can be seen as fitting into a wider class of structured-sparsity inducing models that use prior information on relationships between predictors to enforce different sparsity patterns [25]–[27].

Hierarchical and mixed effect modelling approaches have also been suggested as a means of leveraging pathways information for the simultaneous identification of SNPs or genes within associated pathways. Brenner et al. [28] propose such a method for identifying SNPs in a priori selected candidate pathways by comparing results from multiple studies in a meta-analysis. This approach is similar in motivation to the two-stage methods described above. The method proposed by Wang et al. [29] is closer in spirit to our own, in that it provides measures of pathway significance, and also ranks genes within pathways. Both of these methods however use results from univariate tests of association at each gene variant as input to the models, in contrast to our joint-modelling approach.

Here we describe a method for sparse, pathways-driven SNP selection that extends earlier work using group lasso penalised regression for pathway selection. This latter method was previously shown to offer improved power and specificity for identifying associated pathways, compared with a widely-used alternative [30]. In following sections we describe our method in detail, and demonstrate through simulation that the incorporation of prior information mapping SNPs to gene pathways can boost the power to detect SNPs and genes associated with a quantitative trait. We further describe an application study in which we investigate pathways and genes associated with serum high-density lipoprotein cholesterol (HDLC) levels in two separate cohorts of Asian adults. HDLC refers to the cholesterol carried by small lipoprotein molecules, so called high density lipoproteins (HDLs). HDLs help remove the cholesterol aggregating in arteries, and are therefore protective against cardiovascular diseases [31]. Serum HDLC levels are genetically heritable 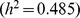 [32]. GWAS studies have now uncovered more than 100 HDLC associated loci (see www.genome.gov/gwastudies, Hindorff et al. [33]). However, considering serum lipids as a whole, variants so far identified account for only 25–30% of the genetic variance, highlighting the limited power of current methodologies to detect hidden genetic factors [34].

## Materials and Methods

This section is organised as follows. We begin by introducing the sparse group lasso (SGL) model for pathways-driven SNP selection, along with an efficient estimation algorithm, for the case of non-overlapping pathways. We then describe a simulation study illustrating superior group (pathway) and variant (SNP) selection performance in the case that the true supporting model is group-sparse. We continue by extending the previous model to the case of overlapping pathways. In principle, we can then solve this model using the estimation algorithm described for the non-overlapping case. However, we argue that this approach does not give us the outcome we require. For this reason we describe a modified estimation algorithm that assumes pathway independence, and demonstrate in a simulation study that this new algorithm is able to identify the correct SNPs and pathways with improved sensitivity and specificity. We next outline a strategy for reducing bias in SNP and pathway selection, and a subsampling procedure that exploits finite sample variation to rank SNPs and genes in order of importance. We test these procedures in a third simulation study using real pathways and genotype data, and conclude that for the range of scenarios tested, our proposed method demonstrates good power and specificity for the detection of associated pathways and genes. We conclude this section with a description of genotypes, phenotypes and pathways used in our application study looking at pathways and genes associated with high-density lipoprotein cholesterol levels in two Asian GWAS cohorts.

### The sparse group lasso model

We arrange the observed values for a univariate quantitative trait or phenotype, measured for *N* unrelated individuals, in an 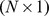 response vector 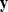. We assume minor allele counts for *P* SNPs are recorded for all individuals, and denote by 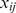 the minor allele count for SNP *j* on individual *i*. These are arranged in an 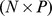 genotype design matrix 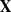. Phenotype and genotype vectors are mean centred, and SNP genotypes are standardised to unit variance, so that 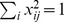, for 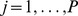.

We assume that all *P* SNPs may be mapped to *L* groups or pathways, 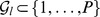, 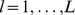, and begin by considering the case where pathways are disjoint or non-overlapping, so that 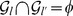 for any 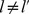. We denote the vector of SNP regression coefficients by 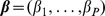, and additionally denote the matrix containing all SNPs mapped to pathway 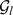 by 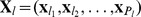, where 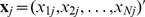, is the column vector of observed SNP minor allele counts for SNP *j*, and 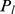 is the number of SNPs in 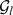. We denote the corresponding vector of SNP coefficients by 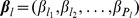.

**Figure 1:**
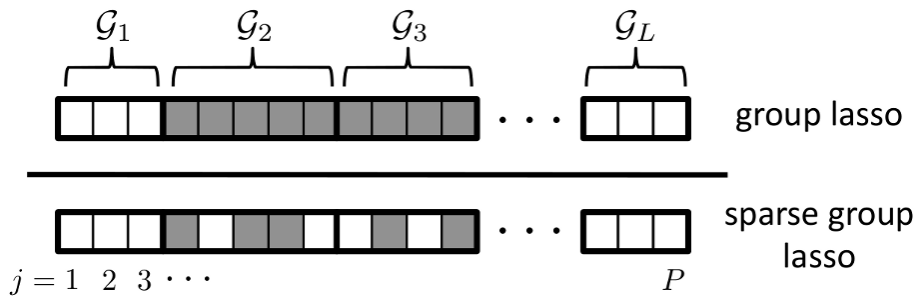
Sparsity patterns enforced by the group lasso and sparse group lasso. The set 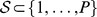 of causal SNPs influencing the phenotype are represented by boxes that are shaded grey. Causal SNPs are assumed to occur within a set 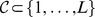 of causal pathways, 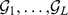. Here 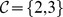. The group lasso enforces sparsity at the group or pathway level only, whereas the sparse group lasso additionally enforces sparsity at the SNP level.

In general, where *P* is large, we expect only a small proportion of SNPs to be ‘causal’, in the sense that they exhibit phenotypic effects. A key assumption in pathways analysis is that these causal SNPs will tend to be enriched within a small set, 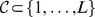, of causal pathways, with 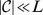, where 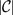 denotes the size (cardinality) of 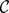. We denote the set of causal SNPs mapping to pathway 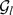 by 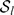, and make the further assumption that most SNPs in a causal pathway are non-causal, so that 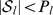, where 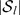 denotes the size (cardinality) of 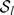. A suitable sparse regression model imposing the required, dual-level sparsity pattern is the sparse group lasso (SGL). We illustrate the resulting causal SNP sparsity pattern in Figure 1, and compare it to that generated by the group lasso (GL), a group-sparse model that we used previously in a sparse regression method to identify gene pathways [17], [30].

With the SGL [20], sparse estimates for the SNP coefficient vector, 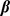 are given by
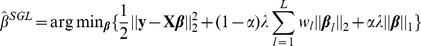
(1)where 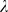 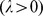 and 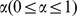 are parameters controlling sparsity, and 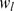 is a pathway weighting parameter that may vary across pathways. (1) corresponds to an ordinary least squares (OLS) optimisation, but with two additional constraints on the coefficient vector, 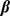, that tend to shrink the size of 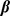, relative to OLS estimates. One constraint imposes a group lasso-type penalty on the size 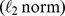 of 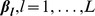. Depending on the values of 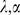 and 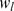, this penalty has the effect of setting multiple pathway SNP coefficient vectors, 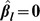, thereby enforcing sparsity at the pathway level. Pathways with non-zero coefficient vectors form the set 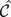 of ‘selected’ pathways, so that
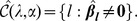
A second constraint imposes a lasso-type penalty on the size 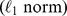 of 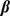. Depending on the values of 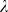 and 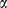, for a selected pathway 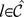, this penalty has the effect of setting multiple SNP coefficient vectors, 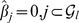, thereby enforcing sparsity at the SNP level within selected pathways. SNPs with non-zero coefficient vectors then form the set 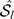 of selected SNPs in pathway *l*, so that
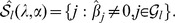
The set of all selected SNPs is given by
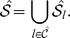
The sparsity parameter 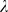 controls the degree of sparsity in 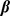, such that the number of pathways and SNPs selected by the model increases as 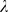 is reduced from a maximal value 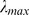, above which 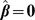. The parameter 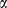 controls how the sparsity constraint is distributed between the two penalties. When 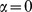, (1) reduces to the group lasso, so that sparsity is imposed only at the pathway level, and all SNPs within a selected pathway have non-zero coefficients. When 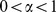, solutions exhibit dual-level sparsity, such that as 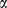 approaches 0 from above, greater sparsity at the group level is encouraged over sparsity at the SNP level. When 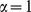, (1) reverts to the lasso, so that pathway information is ignored.

### Model estimation

For the estimation of 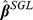 we proceed by noting that the optimisation (1) is convex, and (in the case of non-overlapping groups) that the penalty is block-separable, so that we can obtain a solution using block, or group-wise coordinate gradient descent (BCGD) [35]. A detailed derivation of the estimation algorithm is given in the accompanying Supplementary Information S1, Section 3.

From (S.9) and (S.10), the criterion for selecting a pathway *l* is given by
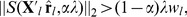
(2)and the criterion for selecting SNP *j* in selected pathway *l* by
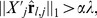
(3)where 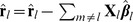 and 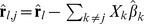 are respectively the pathway and SNP partial residuals, obtained by regressing out the current estimated effects of all other pathways and SNPs respectively. The complete algorithm for SGL estimation using BCGD is presented in Box 1.

**Box 1. SGL-BCGD Estimation Algorithm**

 1. initialise *β*←**0**.

 2. **repeat:** [pathway loop]

for pathway *l* = 1, 2,…, *L*:

if 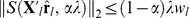

*β_l_*←**0**

else

**repeat:** [SNP loop]

      for 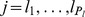:

       if *β_j_* = 0 :

        Newton update 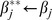 using (S.14)      and (S.12)

       else:

        Newton update 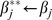 using (S.11)      and (S.12)

       if 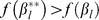:

        

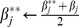

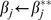

     
**until** convergence of *β_l_* [SNP loop]

**until** convergence of *β* [pathway loop]

 3. 
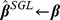

### SGL simulation study 1

We test the hypothesis that where causal SNPs are enriched in a given pathway, pathway-driven SNP selection using SGL will outperform simple lasso selection that disregards pathway information in a simple simulation study. We simulate 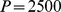 genetic markers for 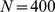 individuals. Marker frequencies for each SNP are sampled independently from a multinomial distribution following a Hardy Weinberg equilibrium frequency distribution. SNP minor allele frequencies are sampled from a uniform distribution 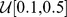. SNPs are distributed equally between 50 non-overlapping pathways, each containing 50 SNPs.

We then test each competing method over 500 Monte Carlo (MC) simulations. At each simulation, a baseline univariate phenotype is sampled from 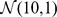. To generate genetic effects, we randomly select 5 SNPs from a single, randomly selected pathway 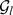, to form the set 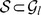 of causal SNPs. Genetic effects are then generated as described in Supplementary Information S1, Section S3.

**Figure 2:**
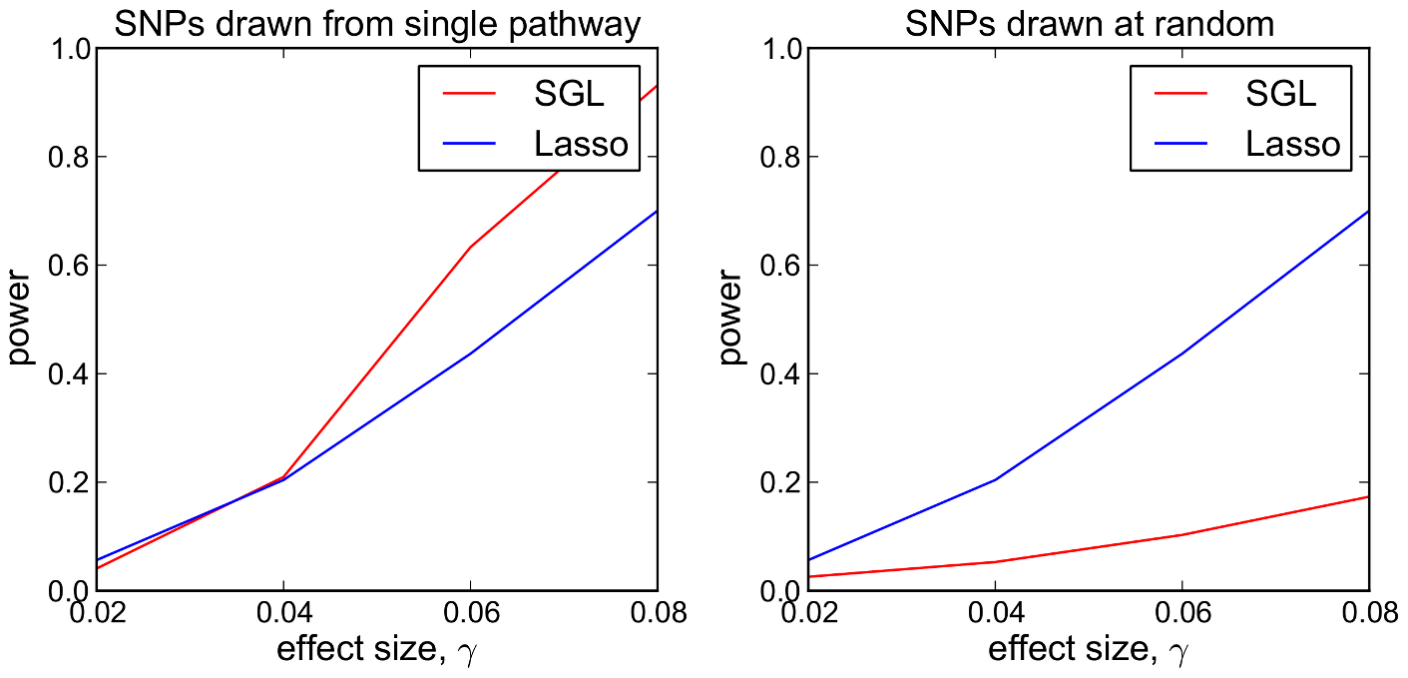
SGL vs Lasso: comparison of power to detect 5 causal SNPs. Each data point represents mean power over 500 MC simulations. *Left:* Causal SNPs drawn from single causal pathway. *Right:* Causal SNPs drawn at random.

To enable a fair comparison between the two methods (SGL and lasso), we ensure that both methods select the same number of SNPs at each simulation. We do this by first obtaining the SGL solution, 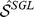, with 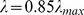 and 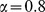, which ensures sparsity at both the pathway and SNP level. We use a uniform pathway weighting vector 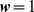. We then compute the lasso solution using coordinate descent over a range of values for the lasso regularisation penalty, 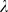, and choose the set
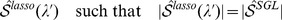
where 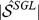 is the number of SNPs previously selected by SGL, and 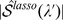 is the number of SNPs selected by the lasso with 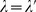. We measure performance as the mean power to detect all 5 causal SNPs over 500 MC simulations, and test a range of genetic effect sizes 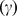 (see Supplementary Information S1, Section S3). In a follow up study, we compare the performance of the two methods in a scenario in which pathways information is uninformative. For this we repeat the previous simulations, but with 5 causal SNPs drawn at random from all 2500 SNPs, irrespective of pathway membership. Results are presented in Figure 2.

Referring to Figure 2, we see that where causal SNPs are concentrated in a single causal pathway (Figure 2 - left), SGL demonstrates greater power (and equivalently specificity, since the total number of selected SNPs is constant), compared with the lasso, above a particular effect size threshold (here 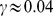). Where pathway information is not important, that is causal SNPs are not enriched in any particular pathway (Figure 2 - right), SGL performs poorly.

**Figure 3:**
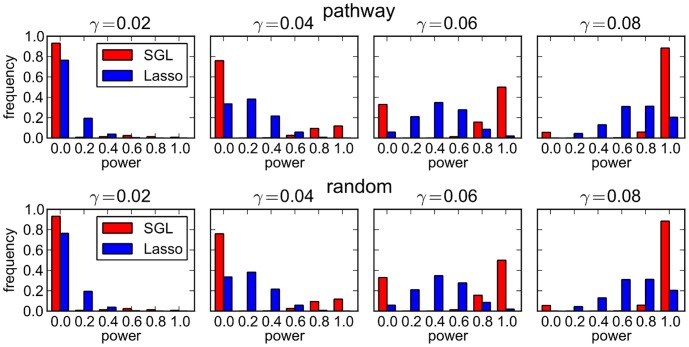
SGL vs Lasso: distribution over 500 MC simulations of power to detect 5 causal SNPs. Each plot represents the power distribution at a single data point in Figure 2. The power distribution is discrete, since each method can identify 0, 1, 2, 3, 4 or 5 causal SNPs, with corresponding power 0, 0.2, 0.4, 0.6, 0.8 or 1.0. *Top row:* Causal SNPs drawn from single causal pathway. *Bottom row:* Causal SNPs drawn at random.

To gain a deeper understanding of what is happening here, we also consider the power distributions across all 500 MC simulations corresponding to each point in the plots of Figure 2. These are illustrated in Figure 3. The top row of plots illustrates the case where causal SNPs are drawn from a single causal pathway. Here we see that there is a marked difference between the two distributions (SGL vs lasso). The lasso shows a smooth distribution in power, with mean power increasing with effect size. In contrast, with SGL the distribution is almost bimodal, with power typically either 0 or 1, depending on whether or not the correct causal pathway is selected. This serves as an illustration of the advantage of pathway-driven SNP selection for the detection of causal SNPs in the case that pathways are important. As previously found by Zhou et al. [6] in the context of rare variants and gene selection, the joint modelling of SNPs within groups gives rise to a relaxation of the penalty on individual SNPs within selected groups, relative to the lasso. This can enable the detection of SNPs with small effect size or low MAF that are missed by the lasso, which disregards pathways information and treats all SNPs equally. Where causal SNPs are not enriched in a causal pathway (bottom row of Figure 3), as expected SGL performs poorly. In this case SGL will only select a SNP where the combined effects of constituent SNPs in a pathway are large enough to drive pathway selection.

Finally, with many pathways methods an adjustment to pathway test statistics is made to account for biases due to variations in pathway size, that is the number of SNPs in a pathway [6]. We explore potential biases using SGL for pathway selection using the simulation framework described above, but this time allowing for varying pathway sizes, ranging from 10 to 200 SNPs. We find no evidence of a pathway size bias (see Supplementary Information S1, Section 5 for further details). We discuss the issue of accounting for pathway size and other potential biases in pathway and SNP selection when using real data in a later section.

### The problem of overlapping pathways

The assumption that pathways are disjoint does not hold in practice, since genes and SNPs may map to multiple pathways (see ‘Pathway mapping’ section below). This means that typically 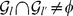 for some 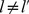. In the context of pathways-driven SNP selection using SGL, this has two important implications. Firstly, the optimisation (1) is no longer separable into groups (pathways), so that convergence using coordinate descent is no longer guaranteed [35]. Secondly, we wish to be able to select pathways independently, and the SGL model as previously described does not allow this. For example consider the case of an overlapping gene, that is a gene that maps to more than one pathway. If a SNP mapping to this gene is selected in one pathway, then it must be selected in each and every pathway containing the mapped gene, so that all pathways mapping to the gene are selected. We instead want to admit the possibility that the joint SNP effects in one pathway may be sufficient to allow pathway selection, while the joint effects in another pathway containing some of the same SNPs do not pass the threshold for pathway selection.

A solution to both these problems is obtained by duplicating SNP predictors in 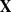, so that SNPs belonging to more than one pathway can enter the model separately [30], [36]. The process works as follows. An expanded design matrix is formed from the column-wise concatenation of the 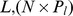 sub-matrices, 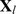, to form the expanded design matrix 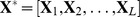 of size 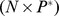, where 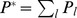. The corresponding 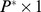 parameter vector, 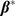, is formed by joining the 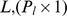 pathway parameter vectors, 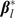, so that 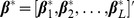. Pathway mappings with SNP indices in the expanded variable space are reflected in updated groups 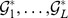. The SGL estimator (1), adapted to account for overlapping groups, is then given by
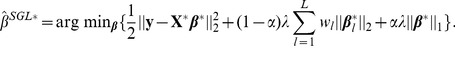
(4)With this overlap expansion, the model is then able to perform pathway and SNP selection in the way that we require, and the corresponding optimisation problem is amenable to solution using the BCGD estimation algorithm described in Box 1. However, for the purpose of pathways-driven SNP selection, the application of this algorithm presents a problem. This arises from the replication of overlapping SNP predictors in each group, 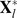, that they occur.

Consider for example the simple situation where there are two pathways, 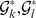, containing sets of causal SNPs 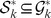 and 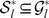 respectively. Here the 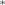 indicates that SNP indices refer to the expanded variable space. We begin by assuming that 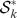 and 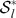 contain the same SNPs, so that in the *unexpanded* variable space, 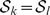.

We then proceed with BCGD by first estimating 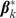. We assume that the correct SNPs are selected, so that 
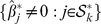
, and 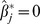 otherwise. For the estimation of 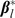, the estimated effect 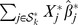, of these overlapping causal SNPs is removed from the regression, through its incorporation in the block residual 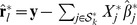. Since no other causal SNPs exist in pathway 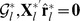, so that the criterion for pathway selection, 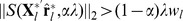 (2) is not met. That is 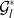 is not selected.

**Figure 4:**
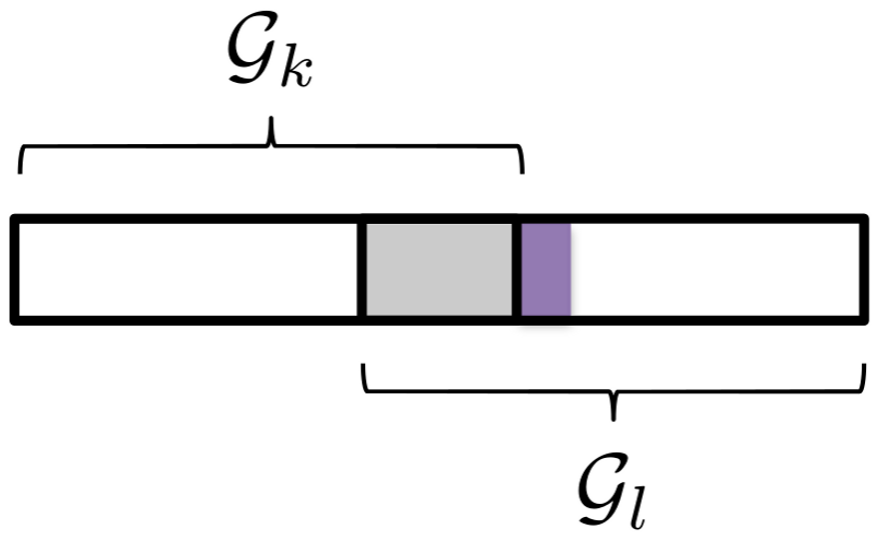
Two pathways with partially overlapping causal SNPs. Causal SNPs (marked in grey) in the set 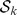 overlap both pathways, so that 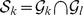. Additional causal SNPs, 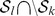, (marked in purple) occur in pathway 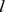 only.

Now consider the case where additional, non-overlapping causal SNPs, possibly with smaller effects, occur in 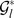, so that in the unexpanded variable space, 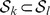. In other words, causal SNPs are *partially overlapping* (see Figure 4). This is the situation for example where multiple causal genes overlap both pathways, but one or more additional causal genes occur in 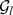. During BCGD pathway 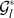 is then less likely to be selected by the model, than would be the case if there were no overlapping SNPs, since once again the effects of overlapping causal SNPs, 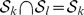, are removed.

For pathways-driven SNP selection, we will argue that we instead require that SNPs are selected in each and every pathway whose joint SNP effects pass a revised pathway selection threshold, irrespective of overlaps between pathways. This is equivalent to the previous pathway selection criterion (2), but with the additional assumption that pathways are independent, in the sense that they do not compete in the model estimation process. We describe a revised estimation algorithm under the assumption of pathway independence below.

We justify the strong assumption of pathway independence with the following argument. In reality, we expect that multiple pathways may simultaneously influence the phenotype, and we also expect that many such pathways will overlap, for example through their containing one or more ‘hub’ genes, that overlap multiple pathways [37], [38]. By considering each pathway independently, we aim to maximise the sensitivity of our method to detect these variants and pathways. In contrast, without the independence assumption, a competitive estimation algorithm will tend to pick out one from each set of similar, overlapping pathways, and miss potentially causal pathways and variants as a consequence. We illustrate this idea in the simulation study in the following section. One potential concern is that by not allowing pathways to compete against each other, specificity may be reduced, since too many pathways and SNPs may be selected. We discuss the issue of specificity further in the context of results from the simulation study.

A detailed derivation of the SGL model estimation algorithm under the independence assumption is given in Supplementary Information S1, Section 2. The main results are that the pathway (2) and SNP (3) selection criteria become
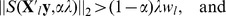


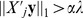
(5)respectively. The key difference is that partial derivatives 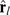 and 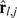 are replaced by 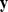, that is each pathway is regressed against the phenotype vector 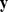. This means that there is no block coordinate descent stage in the estimation, so that the revised algorithm utilises only coordinate gradient descent within each selected pathway. For this reason we use the acronym SGL-CGD for the revised algorithm, and SGL-BCGD for the previous algorithm using block coordinate gradient descent. The new algorithm is described in Box 2.

**Box 2. SGL-CGD Estimation Algorithm for Overlapping Pathways**

 1. initialise 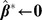.

 2. for pathway *l* = 1, 2,…, *L*:

   if 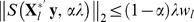

    

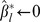

   else

    
**repeat:** [CGD (SNP) loop]

     for 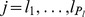:

      if 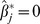 :

       Newton update 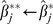 using (S.21) and (S.12)

      else:

       Newton update 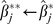 using (S.20) and (S.12)

      if 
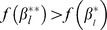
:

       

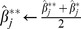

      

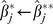

    
**until convergence**

 3. 
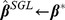

Finally, we note that for SNP selection we are interested only in the set 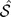 of selected SNPs in the unexpanded variable space, and not the set 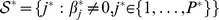. Since, under the independence assumption, the estimation of each 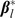 does not depend on the other estimates, 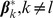, we do not need to record separate coefficient estimates for each pathway in which a SNP is selected. Instead we need only record the set 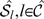 of SNPs selected in each selected pathway. This has a useful practical implication, since we can avoid the need for an expansion of 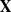 or 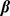, and simply form the complete set of selected SNPs as
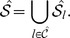



### SGL simulation study 2

We now explore some of the issues raised in the preceding section, specifically the potential impact on pathway and SNP selection power and specificity of treating the pathways as independent in the SGL estimation algorithm. We do this in a simulation study in which we simulate overlapping pathways. The simulation scheme is specifically designed to highlight differences in pathway and SNP selection with the independence assumption (using the SGL-CGD estimation algorithm in Box 2) and without it (using the standard SGL estimation algorithm in Box 1).

**Figure 5:**
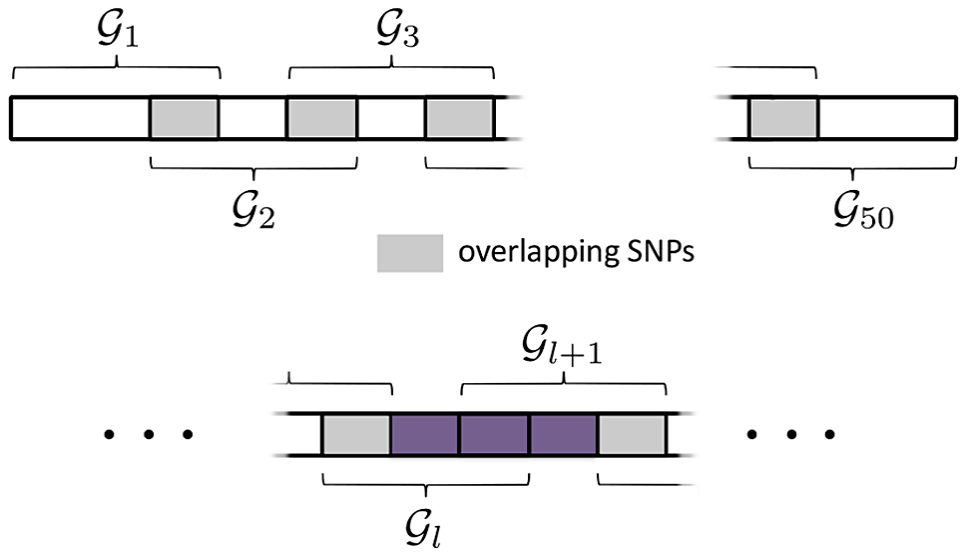
SGL Simulation Study with overlapping pathways. *Top:* Illustration of pathway overlap scheme. The are 30 SNPs in each pathway. Pathways 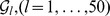 overlap each adjacent pathway by 10 SNPs. *Bottom:* Causal SNPs from adjacent pathways, 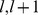 are randomly selected from the region marked in purple, ensuring that SNPs in 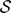 overlap a maximum of two pathways.

SNPs with variable MAF are simulated using the same procedure described in the previous simulation study, but this time SNPs are mapped to 50 *overlapping* pathways, each containing 30 SNPs. Each pathway overlaps any adjacent (by pathway index) pathway by 10 SNPs. This overlap scheme is illustrated in Figure 5 (top).

As before we consider a range of overall genetic effect sizes, 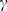. A total of 2000 MC simulations are conducted for each effect size. At MC simulation 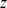, we randomly select two adjacent pathways, 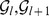 where 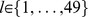. From these two pathways we randomly select 10 SNPs according to the scheme illustrated in Figure 5 (bottom). This ensures that causal SNPs overlap a minimum of 1, and a maximum of 2 pathways, with 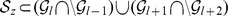. The true set of causal pathways, 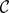, is then given by 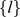, 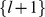 or 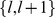 (although simulations where 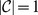 will be extremely rare). Genetic effects on the phenotype are generated as described previously (Supplementary Information S1, Section S3).

SNP coefficients are estimated for each algorithm, SGL-BCGD and SGL-CGD, using the same regularisation with 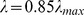 and 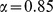 for both.

The average number of pathways and SNPs selected by SGL-BCGD and SGL-CGD across all 2000 MC simulations is reported in Table 1. As expected, for both models, the number of selected variables (pathways or SNPs) increases with decreasing effect size, as the number of pathways close to the selection threshold set by 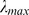 increases.

**Table 1:** Simulation study 2: Mean number of pathways and SNPs selected by each model at each effect size, *γ*, across 2000 MC simulations.

		*γ*					
		0.02	0.04	0.06	0.08	0.1	0.12
pathways	SGL-CGD	5.8	5.9	5.4	4.8	3.9	3.2
	SGL-BCGD	5.8	5.9	5.4	4.8	3.9	3.2
SNPs	SGL-CGD	26.6	27.0	24.8	22.2	18.5	15.3
	SGL-BCGD	28.8	29.3	26.7	23.6	19.4	15.8

For each model, at MC simulation 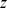 we record the pathway and SNP selection power, 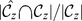 and 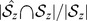 respectively. Since the number of selected variables can vary slightly between the two models, we also record false positive rates (FPR) for pathway and SNP selection as 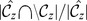 and 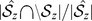 respectively.

**Figure 6:**
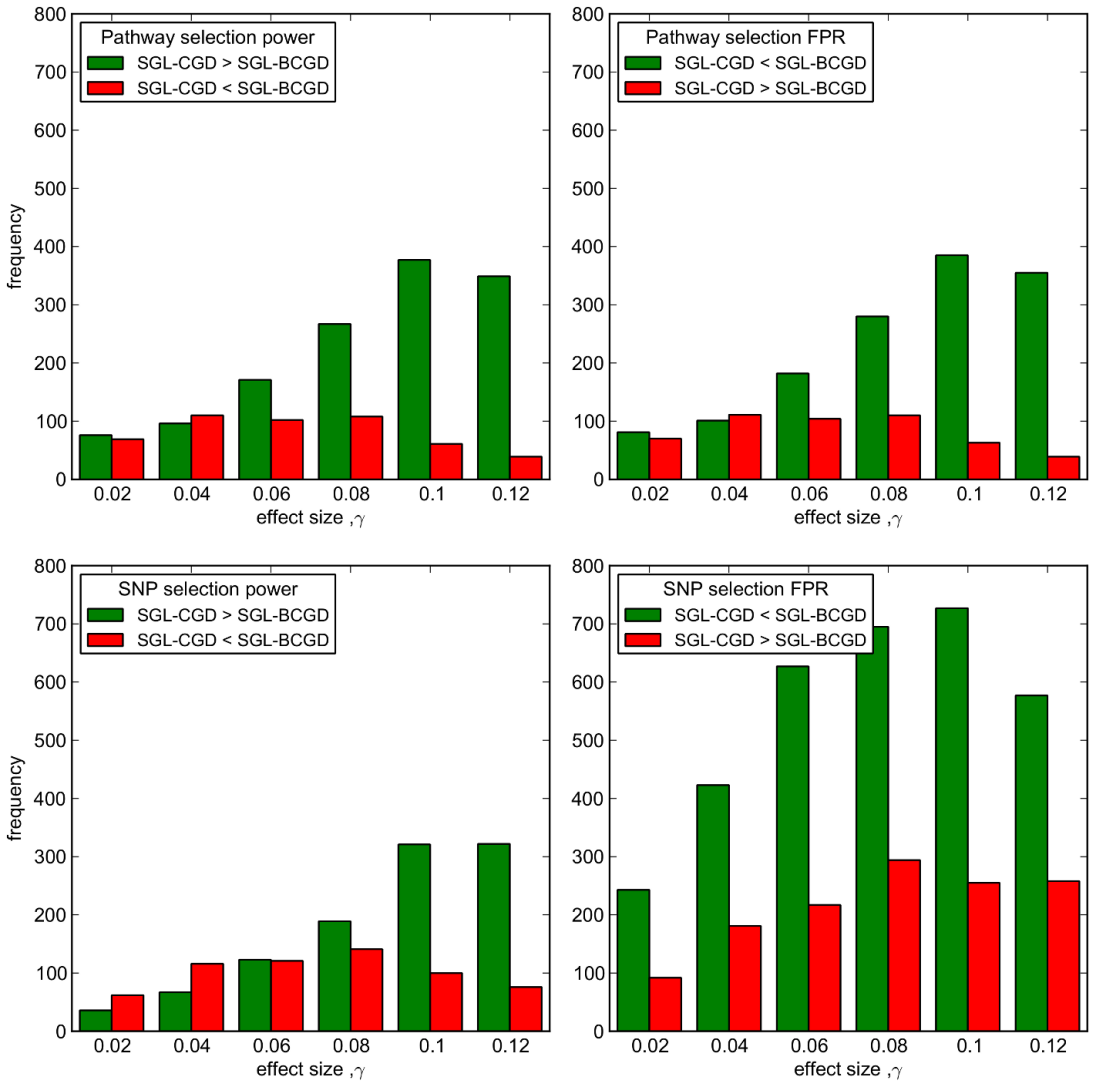
SGL-CGD vs SGL-BCGD performance, measured across 2000 MC simulations. *Top row:* Pathway selection performance. (Left) green bars indicate the number of MC simulations where SGL-CGD has greater pathway selection power than SGL. Red bars indicate where SGL-BCGD has greater power than SGL-CGD. (Right) green bars indicate the number of MC simulations where SGL-CGD has a lower FPR than SGL. Red bars indicate the opposite. *Bottom row:* As above, but for SNP selection performance.

The large possible variation in causal SNP distributions, causal SNP MAFs etc. makes a comparison of mean power and FPR between the two methods somewhat unsatisfactory. For example, depending on effect size, a large number of simulations can have either very high, or very low pathway and SNP selection power, masking subtle differences in performance between the two methods. Since we are specifically interested in establishing the relative performance of the two methods, we instead illustrate the number of simulations at which one method outperforms the other across all 2000 MC simulations, and show this in Figure 6. In this figure, the number of simulations in which SGL-CGD outperforms SGL, i.e. where SGL-CGD power>SGL-BCGD power, or SGL-CGD FPR<SGL-BCGD FPR, are shown in green. Conversely, the number of simulations where SGL-BCGD outperforms SGL-CGD are shown in red.

We first consider pathway selection performance (top row of Figure 6). For both methods, the same number of pathways are selected on average, across all effect sizes (Table 1). At low effect sizes, there is no difference in performance between the two methods for the large majority of MC simulations, and where there is a difference, the two methods are evenly balanced. As with SGL Simulation Study 1, this is the region (with 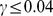) where pathway selection fairs no better than chance. With 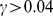, SGL-CGD consistently outperforms SGL, both in terms of pathway selection sensitivity and control of false positives (measured by FPR).

To understand why, we turn to SNP selection performance (bottom row of Figure 6). At small effect sizes 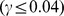, in the small minority of simulations where the correct pathways are identified, SGL-BCGD tends to demonstrate greater power than SGL-CGD (Figure 6 bottom left). However, this is at the expense of lower specificity (Figure 6 bottom right). These difference are due to the slightly larger number of SNPs selected by SGL-BCGD (see Table 1), which in turn is due to the ‘screening out’ of previously selected SNPs from the adjacent causal pathway during BCGD, as described previously. This results in the selection of a larger number of SNPs when any two overlapping pathways are selected by the model. In the case where two causal pathways are selected, SNP selection power is then likely to be higher, although at the expense of a greater number of false positives.

When pathway effects are just on the margin of detectability 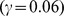, SGL-CGD is more often able to select both causal pathways, although this doesn't translate into increased SNP selection power. This is most likely because at this effect size neither model can detect SNPs with low MAF, so that SGL-CGD is detecting the same (overlapping) SNPs in both causal pathways. Note that once again SGL-BCGD typically has a higher FPR than SGL-CGD, since more SNPs are selected from non-causal pathways.

As the effect size increases, the number of simulations in which SGL-CGD outperforms SGL-BCGD for SNP selection power grows, paralleling the former method's enhanced pathway selection power. This is again a demonstration of the screening effect with SGL-BCGD described previously. This means that SGL-CGD is more often able to select both causal pathways, and to select additional causal SNPs that are missed by SGL. These additional SNPs are likely to be those with lower MAF, for example, that are harder to detect with SGL, once the effect of overlapping SNPs are screened out during estimation using BCGD. Interestingly, as before SGL-CGD continues to exhibit lower false positive rates than SGL. This suggests that, with the simulated data considered here, the independence assumption offers better control of false positives by enabling the selection of causal SNPs in each and every pathway to which they are mapped. In contrast, where causal SNPs are successively screened out during the estimation using BCGD, too many SNPs with spurious effects are selected.

The relative advantage of SGL-CGD over SGL-BCGD on all performance measures starts to decrease around 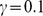, as SGL-BCGD becomes better able to detect all causal pathways and SNPs, irrespective of the screening effect.

### Pathway and SNP selection bias

One issue that must be addressed is the problem of selection bias, by which we mean the tendency of SGL to favour the selection of particular pathways or SNPs under the null, where no SNPs influence the phenotype. Possible biasing factors include variations in pathway size or varying patterns of SNP-SNP correlations and gene sizes. Common strategies for bias reduction include the use of dimensionality reduction techniques and permutation methods [39]–[42].

In earlier work we described an adaptive weight-tuning strategy, designed to reduce selection bias in a group lasso-based pathway selection method [30]. This works by tuning the pathway weight vector, 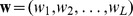, so as to ensure that pathways are selected with equal probability under the null. This strategy can be readily extended to the case of dual-level sparsity with the SGL.

Our procedure rests on the observation that for pathway selection to be unbiased, each pathway must have an equal chance of being selected. For a given 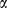, and with 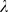 tuned to ensure that a single pathway is selected, pathway selection probabilities are then described by a uniform distribution, 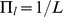, for 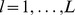. We proceed by calculating an empirical pathway selection frequency distribution, 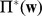, by determining which pathway will first be selected by the model as 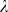 is reduced from its maximal value, 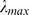, over multiple permutations of the response, 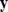. This process is described in detail in Supplementary Information S1, Section 4. We note that alternative methods for the construction of ‘null’ distributions, for example by permuting genotype labels, have been used in existing pathways analysis methods [6]. In the present context we choose to permute phenotype labels in order to preserve LD structure, since we expect this to be a significant source of bias with our data.

Our iterative weight tuning procedure then works by applying successive adjustments to the pathway weight vector, 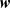, so as to reduce the difference, 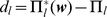, between the unbiased and empirical (biased) distributions for each pathway. At iteration 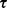, we compute the empirical pathway selection probability distribution 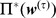, determine 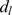 for each pathway, and then apply the following weight adjustment
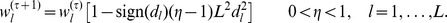
The parameter 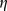 controls the maximum amount by which each 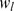 can be reduced in a single iteration, in the case that pathway *l* is selected with zero frequency. The square in the weight adjustment factor ensures that large values of 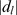 result in relatively large adjustments to 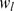. Iterations continue until convergence, where 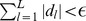.

Note that when multiple pathways are selected by the model, the expected pathway selection frequency distribution under the null will not be uniform. This is because pathways overlap, so that selection frequencies will reflect the complex distribution of overlapping genes, as indeed will unbiased empirical selection frequencies. We have shown previously that this adaptive weight-tuning procedure gives rise to substantial gains in sensitivity and specificity with regard to pathway selection [30].

### Ranking variables

With most variable selection methods, a choice for the regularisation parameter, 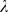, must be made, since this determines the number of variables selected by the model. Common strategies include the use of cross validation to choose a 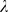 value that minimises the prediction error between training and test datasets [43]. One drawback of this approach is that it focuses on optimising the *size* of the set, 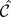, of selected pathways (more generally, selected variables) that minimises the cross validated prediction error. Since the variables in 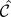 will vary across each fold of the cross validation, this procedure is not in general a good means of establishing the importance of a unique set of variables, and can give rise to the selection of too many variables [44], [45]. For the lasso, alternative approaches, based on data subsampling or bootstrapping have been shown to improve model consistency, in the sense that the correct model is selected with a high probability [45]–[47]. These methods work by recording selected variables across multiple subsamples of the data, and forming the final set of selected variables either as the intersection of variables selected at each model fit, or by assessing variable selection frequencies. Examples of the use of such approaches can be found in a number of recent gene mapping studies involving model selection using either the lasso or elastic net [9], [19], [44], [48]. Motivated by these ideas, we adopt a resampling strategy in which we calculate pathway, gene and SNP selection frequencies by repeatedly fitting the model over *B* subsamples of the data, at fixed values for 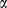 and 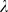. Each random subsample of size 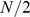 is drawn without replacement. Our motivation here is to exploit knowledge of finite sample variability obtained by subsampling, to achieve better estimates of a variable's importance. With this approach, which in some respects resembles the ‘pointwise stability selection’ strategy of Meinshasen and Bühlmann [45], selection frequencies provide a direct measure of confidence in the selected variables in a finite sample. This resampling strategy also allows us to rank pathways, genes and SNPs in order of their strength of association with the phenotype, so that we expect the true set of causal variables to achieve a high ranking, whereas non-causal variables will be ranked low.

There have however been suggestions that the use of lasso-type penalties in combination with a subsampling approach can be problematic when applied to GWAS data, where there is widespread correlation between SNPs [49]. This is due to the lasso's tendency to single out different SNPs within an LD block from subsample to subsample, depressing variable selection frequencies for groups of SNPs with high LD. Possible remedies include the use of grouping or sliding-window type strategies, so that neighbouring SNPs in high LD are added to the set of selected SNPs at each subsample. We test the relative performance of these different strategies in a final simulation study described in the next section.

For pathway ranking, we denote the set of selected pathways at subsample *b* by
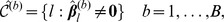
where 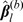 is the estimated SNP coefficient vector for pathway *l* at subsample *b*. The selection probability for pathway *l* measured across all *B* subsamples is then
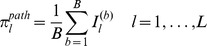
where the indicator function, 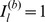 if 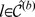, and 0 otherwise. Pathways are ranked in order of their selection probabilities, 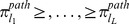.

For SNP ranking, we denote the set of SNPs selected at subsample *b* (in the unexpanded variable space) by 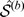, and further denote the set of all SNPs within a specified LD threshold, *r* of SNPs in 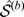 by 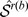 (including SNPs in 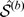). We use an 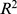 correlation coefficient 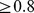 for this threshold. Using the same procedure as for pathway ranking, we then obtain two possible expressions for the selection probability of SNP *j* across *B* subsamples as
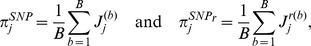
where the indicator functions, 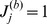 if 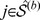, and 0 otherwise; and 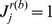 if 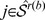, and 0 otherwise.

Finally, for gene ranking we denote the set of selected genes to which the SNPs in 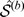 are mapped by 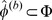, where 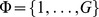 is the set of gene indices corresponding to all *G* mapped genes. An expression for the selection probability for gene *g* is then
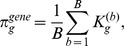
where the indicator function 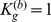 if 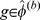, and 0 otherwise. SNPs and genes are ranked in order of their respective selection frequencies.

Software implementing the methods described here, together with sample data is available at http://www2.imperial.ac.uk/~gmontana/psrrr.htm.

### Simulation study 3

We evaluate the performance of the above strategies for ranking pathways, SNPs and genes in a final simulation study. For this study we use real genotype and pathways data so that we can gauge variable selection performance in the presence of LD, and variations in the distribution of gene and pathway sizes and of overlaps. For these simulations we use genome-wide SNP data from the ‘SP2’ dataset and map SNPs to pathways from the KEGG pathways database (see following sections for further details). This dataset comprises 1,040 individuals, each genotyped at 542,297 SNPs, of which 75,389 SNPs can be mapped to 4,734 genes and 185 pathways with a mean pathway size of 1,080 SNPs.

We test a number of different scenarios in which we vary the numbers of causal SNPs and SNP effect sizes. For each scenario we perform 400 MC simulations. For each MC simulation we select *k* causal SNPs at random from a single randomly selected causal pathway. Note however that because pathways can overlap, different numbers of causal SNPs (up to a maximum number *k*) may overlap more than one pathway. We then generate a quantitative phenotype in which we control the per-locus effects size, 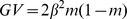, where 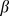 is the proportionate change in phenotype per causal allele, and *m* is the locus minor allele frequency. *GV* is then the total proportion of trait variance attributable to each causal locus under an additive model, and under Hardy-Weinberg equilibrium [50]. We also report the total variance, *TV*, which is the proportion of trait variance attributable to all causal loci.

Using contemporaneous GWAS data, Park et al. [50], report values for *GV* ranging from 0.0004 to 0.02 for three complex traits (height, Crohns disease and breast, prostate and colorectal (BPC) cancers), although clearly only the largest studies will have sufficient power to identify the smallest genetic effects. They additionally produce estimates ranging from 67 to 201 for the total number of susceptibility loci using these effect sizes, with corresponding values for *TV* ranging from 0.1 to 0.36 (95% CI). It is interesting to note that for certain diseases there is also evidence for polygenic modes of inheritance involving many thousands of SNPs with small effects [51]. While it is currently impossible to translate findings from these and other GWAS into an understanding of how causal SNPs might be distributed within putative causal pathways, we are guided in part by these reported values in constructing our six simulation test scenarios, which are listed in Table 2. These are designed to cover cases where the number of causal SNPs is relatively small 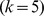, or large 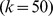 relative to pathway size, and to test cases where the proportion of trait variance explained by causal SNPs spans a realistic range.

**Table 2:** Simulation study 3: Six scenarios tested.

scenario	*k*	*GV*	*TV*	mean # selected SNPs at each subsample	mean # ranked SNPs across all simulations
(a)	5	0.005	0.03	85	4856
(b)	5	0.01	0.05	71	4170
(c)	5	0.05	0.2	43	483
(d)	50	0.001	0.1	65	3803
(e)	50	0.005	0.2	57	903
(f)	50	0.01	0.4	56	496

For simplicity, we set the regularisation parameter 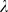 to be very close to 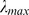, to ensure that a single pathway is selected at each of the 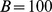 subsamples generated for each simulation. We set 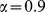 and characterise the resulting SNP sparsity in the final two columns of Table 2. At each MC simulation, all causal SNPs used to generate the phenotype are removed from the genotype data prior to model fitting.

**Figure 7:**
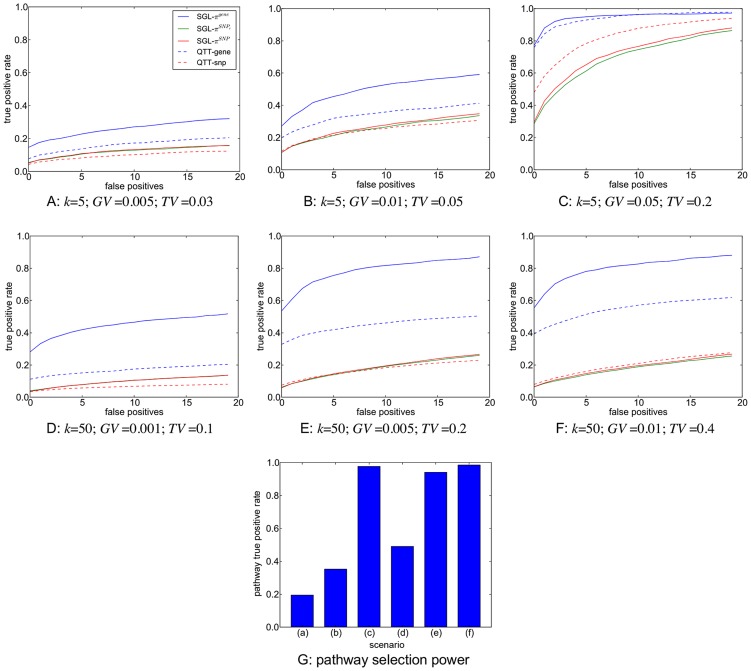
A–F: SNP and gene ranking performance for the six different scenarios described in Table 2. Plots show mean true positive rates over 400 MC simulations for each scenario. Three different subsample ranking methods (solid lines) are used for SGL, as described in the previous section. SNP and gene ranking performance obtained by ranking p-values from a univariate, regression-based quantitative trait test (QTT - dashed lines) are shown for comparison. Definitions for true positive rates and number of false positives are described in the main text. G: Pathway selection performance for each scenario. True positive rates represent the proportion of simulations in which the correct causal pathway is selected.

In Figure 7(g) we present the proportion of subsamples (across all MC simulations) in which the correct causal pathway is selected, for each of the scenarios described in Table 2. Since pathways overlap, a causal pathway is here defined as any pathway containing one or more causal SNPs. Since only one pathway is selected at each subsample, true positive rates for each scenario represent the mean number of subsamples in which a causal pathway is selected, across all MC simulations.

In Figure 7(a)–(f) we present results for SNP and gene ranking performance using SGL-CGD in combination with our resampling-based ranking strategy, using the three different selection frequency measures, 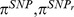 and 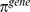, described in the previous section. For SNP rankings, since actual causal SNPs used to generate phenotypes are removed, true positives are defined as selected SNPs that tag at least one causal SNP with an 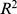 coefficient 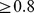. False positives are selected SNPs which do not tag any causal SNP. For gene rankings, causal genes are defined as those that map to a true causal SNP. True positives are then selected causal genes, and false positives are selected non-causal genes. Since the number of ranked variables varies across simulations, mean true positive rates across all simulations are plotted against the number of selected false positives for each scenario. Thus, for a particular simulation, if the highest ranking false positive is at rank *z*, then the number of true positives is 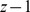, and the true positive *rate* for a single false positive is the proportion of true causal variables (SNPs or genes) that are tagged by these 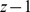 selected variables. SNP and gene rankings using a univariate, regression-based quantitative trait test (QTT) for association are also presented for comparison. For SNP rankings, variables are ranked by their QTT p-value. For gene rankings, SNPs are first mapped to genes, and genes are then ranked by their smallest associated SNP p-value. SNP to gene mappings for all methods are determined in the same way as for mapping SNPs to pathways, that is SNPs are mapped to genes within 10 kbp upstream or downstream of the SNP in question (see ‘Pathway mapping’ section below).

It is immediately apparent that the best performance, both in terms of power and control of false positives, is obtained by grouping selected SNPs into genes, that is when ranking by gene selection frequency, 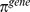. As described elsewhere [49], simple ranking by SNP selection frequency 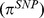 gives poor results, even if we extend SNP selection to include nearby SNPs in strong LD with selected variants 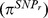. A notable feature of our method is highlighted by comparing scenarios (c) and (e). In scenario (c), the genetic variance explained by each causal locus is relatively high, and gene ranking performance for both QTT and SGL is very good. For scenario (e), the proportion of total phenotypic variance explained by causal loci is the same as that in (c) 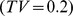, but in the former relatively small genetic effects are distributed across a larger number of causal loci 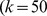 vs. 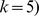. Pathway selection power is maintained by SGL for both scenarios, and SGL is also able to maintain superior gene ranking performance with relatively high power and good control of false positives compared to QTT where performance is poor. Also of interest is the fact that SGL gene ranking performance is able to outperform QTT SNP and gene ranking, even at the smallest per-locus effect sizes (measured by *GV* - scenarios (a) and (d)), where pathway selection performance is relatively low. Note that in some cases (most notably in scenario (a)), SGL SNP and gene ranking power can exceed pathway selection power. This is because true positive SNPs or genes may be ranked higher than false positives, even in the case that a causal pathway is selected in relatively few subsamples. Indeed this ability to distinguish true from false positives in variable rankings at low signal to noise thresholds is one of the attractive features of our subsampling approach.

We conclude from this simulation study that SGL in combination with gene ranking using our proposed subsampling approach is able to demonstrate good power and specificity over a range of scenarios using real genotype and pathways data. We next use this approach in an application study which we describe in the remainder of this article.

### Subjects, genotypes and phenotypes

Our application study using pathways-driven SNP selection to search for pathways and genes associated with variation in serum high-density lipoprotein cholesterol levels is carried out using data from two separate cohorts of Asian adults. These datasets have previously been used to search for novel variants associated with type 2 diabetes mellitus (T2D) in Asian populations. The first (discovery) cohort is from the Singapore Prospective Study Program, hereafter referred to as ‘SP2’, and the second (replication) dataset is from the Singapore Malay Eye Study or ‘SiMES’. Detailed information on both datasets can be found in [52], but we briefly outline some salient features here.

Both datasets comprise whole genome data for T2D cases and controls, genotyped on the Illumina HumanHap 610 Quad array. For the present study we use controls only, since variation in lipid levels between cases and controls can be greater than the variation within controls alone. The use of both cases and controls in our analysis might then lead to a confounded analysis, where any associations could be linked to T2D status or some other spurious factor.

A full investigation of population stratification for the SP2 dataset was carried out for the original GWAS study using PCA with 4 panels from the International Hapmap Project and the Singapore Genome Variation Project, to ensure that this dataset contained only ethnic Chinese [52]–[54]. The SiMES dataset comprises ethnic Malays, and shows some evidence of cryptic relatedness between samples. For this reason, the first two principal components of a PCA for population structure are used as covariates in our analysis of this dataset. Again full details of the stratification analysis can be found in [52] and associated Supplementary Information.

A summary of information pertaining to genotypes for each dataset, both before and after imputation and pathway mapping, is given in Table 3, along with a list of phenotypes and covariates.

**Table 3:** Genotype and phenotype information corresponding to the SP2 and SiMES datasets used in the study.

	SP2	Simes
Sample size	*N* = 1,040	*N* = 1,099
**Genotypes**		
*Before imputation*		
SNPs available for analysis(1)	542,297	557,824
SNPs with missing genotypes(2)	152,372	282,549
*Post imputation*		
SNPs available for analysis(3)	492,639	515,503
**Phenotypes/covariates**		
quantitative trait (phenotype)(4)	HDLC	HDLC
covariates	gender, age, age^2^,	gender, age, age^2^,
	BMI(5)	BMI, PC1, PC2(6)

(1) after first round of quality control [52] and removal of monomorphic SNPs.

(2) maximum 5% missing rate per SNP.

(3) after imputation and removal of SNPs with 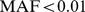.

(4) mg/dL.

(5) body mass index 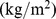.

(6) principal components relating to cryptic relatedness.

### Genotype imputation

After the initial round of quality control, genotypes for both datasets have a maximum SNP missingness of 5%. Since our method cannot handle missing values, we perform ‘missing holes’ SNP imputation, so that all missing SNP calls are estimated against a reference panel of known haplotypes.

SNP imputation proceeds in two stages. First, imputation requires accurate estimation of haplotypes from diploid genotypes (phasing). This is performed using SHAPEIT v1 (http://www.shapeit.fr). This uses a hidden Markov model to infer haplotypes from sample genotypes using a map of known recombination rates across the genome [55]. The recombination map must correspond to genotype coordinates in the dataset to be imputed, so we use recombination data from HapMap phase II, corresponding to genome build NCBI b36 (http://hapmap.ncbi.nlm.nih.gov/downloads/recombination/2008-03_rel22_B36/).

Following the primary phasing stage, SNP imputation is performed using IMPUTE v2.2.2 (http://mathgen.stats.ox.ac.uk/impute/impute_v2.html). IMPUTE uses a reference panel of known haplotypes to infer unobserved genotypes, given a set of observed sample haplotypes [56]. The latest version (IMPUTE 2) uses an updated, efficient algorithm, so that a custom reference panel can be used for each study haplotype, and for each region of the genome, enabling the full range of reference information provided by HapMap3 [57] to be used. Following IMPUTE 2 guidelines, we use HapMap3 reference data corresponding to NCBI b36 (http://mathgen.stats.ox.ac.uk/impute/data_download_hapmap3_r2.html) which includes haplotype data for 1,011 individuals from Africa, Asia, Europe and the Americas. SNPs are imputed in 5MB chunks, using an effective population size (*Ne*) of 15,000, and a buffer of 250 kb to avoid edge effects, again as recommended for IMPUTE 2.

### Pathway mapping

Pathways GWAS methods rely on prior information mapping SNPs to functional networks or pathways. Since pathways are typically defined as groups of interacting genes, SNP to pathway mapping is a two-part process, requiring the mapping of genes to pathways, and of SNPs to genes. A consistent strategy for this mapping process has however yet to be established, a situation compounded by a lack of agreement on what constitutes a pathway in the first place [58].

The number and size of databases devoted to classifying genes into pathways is growing rapidly, as is the range and diversity of gene interactions considered (see for example http://www.pathguide.org/). Databases such as those provided by KEGG (http://www.genome.jp/kegg/pathway.html), Reactome (http://www.reactome.org/) and Biocarta (http://www.biocarta.com/) classify pathways across a number of functional domains, for example apoptosis, cell adhesion or lipid metabolism; or crystallise current knowledge on specific disease-related molecular reaction networks. Strategies for pathways database assembly range from a fully-automated text-mining approach, to that of careful curation by experts. Inevitably therefore, there is considerable variation between databases, in terms of both gene coverage and consistency [59], so that the choice of database(s) will itself influence results in pathways GWAS.

**Figure 8:**
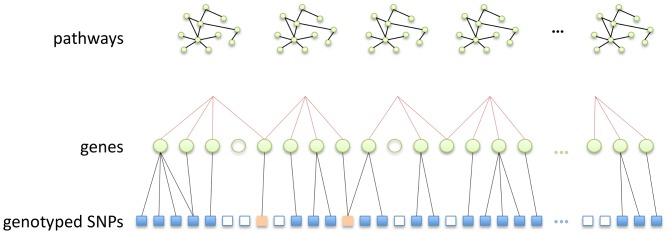
Schematic illustration of the SNP to pathway mapping process. (i) Genes (green circles) are mapped to pathways using information on gene-gene interactions (top row), obtained from a gene pathways database. Many genes do not map to any known pathway (unfilled circles). Also, some genes may map to more than one pathway. (ii) Genes that map to a pathway are in turn mapped to genotyped SNPs within a specified distance. Many SNPs cannot be mapped to a pathway since they do not map to a mapped gene (unfilled squares). Note SNPs may map to more than one gene. Some SNPs (orange squares) may map to more than one pathway, either because they map to multiple genes belonging to different pathways, or because they map to a single gene that belongs to multiple pathways.

The mapping of SNPs to genes adds a further layer of complexity, since although many SNPs may occur within gene boundaries, on a typical GWAS array the vast majority of SNPs will reside in inter-genic regions. In an attempt to include variants potentially residing in functionally significant regions lying outside gene boundaries, SNPs may be mapped to nearby genes using various distance thresholds. Various values for SNP to gene mapping distances, measured in thousands of nucleotide base pairs (kb), have been suggested in the literature, ranging from mapping SNPs to genes only if they fall within a specific gene, to the attempt to encompass upstream promoters and enhancers by extending the range to 10, 20 or even 500 kb and beyond [18], [39], [58]. This process is illustrated schematically in Figure 8. Notable features of the SNP to pathway mapping process include the fact that genes (and therefore SNPs) may map to more than one pathway, and also that many SNPs and genes do not currently map to any known pathway [7].

**Figure 9:**
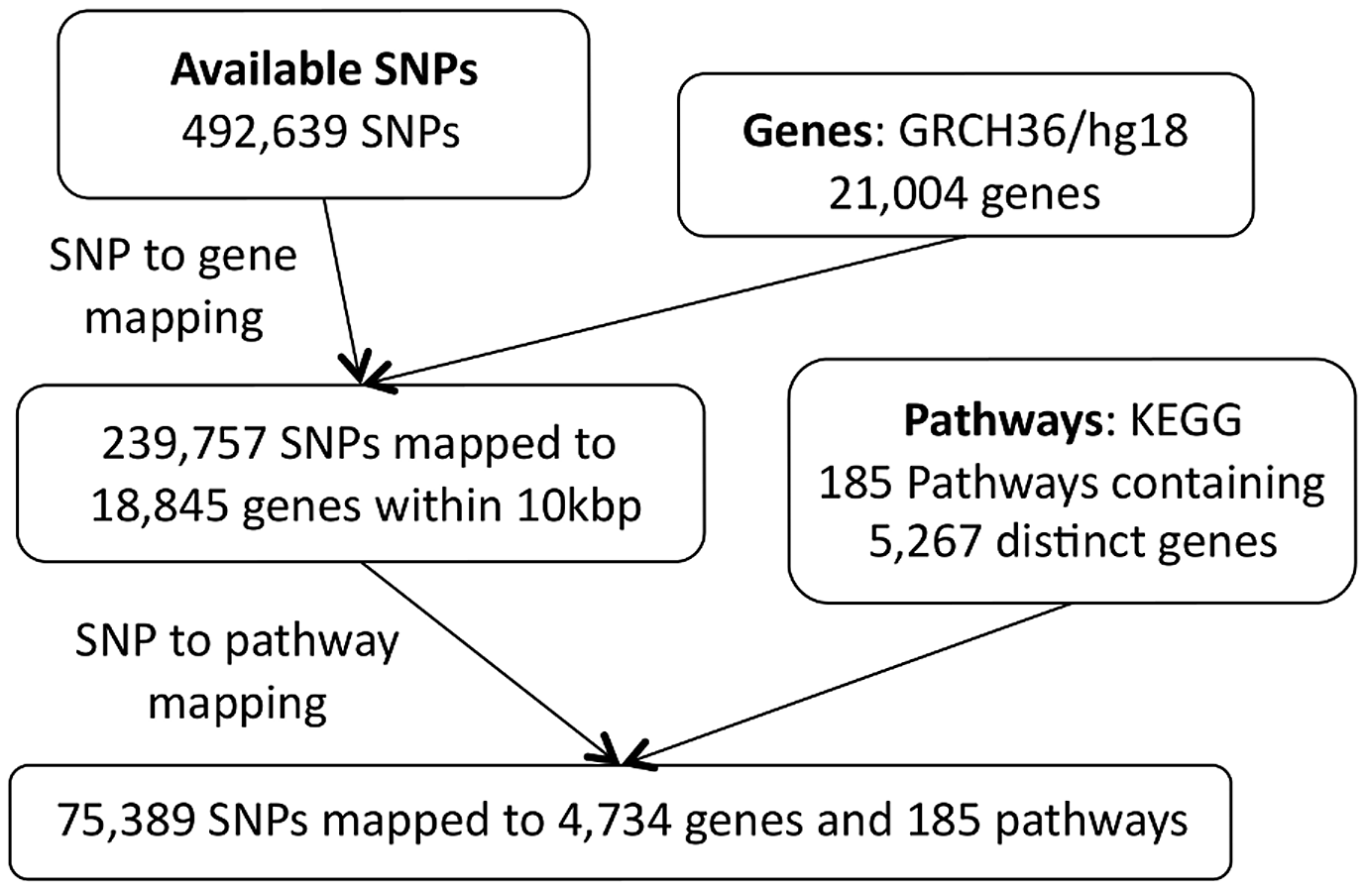
SP2 dataset: SNP to pathway mapping.

**Figure 10:**
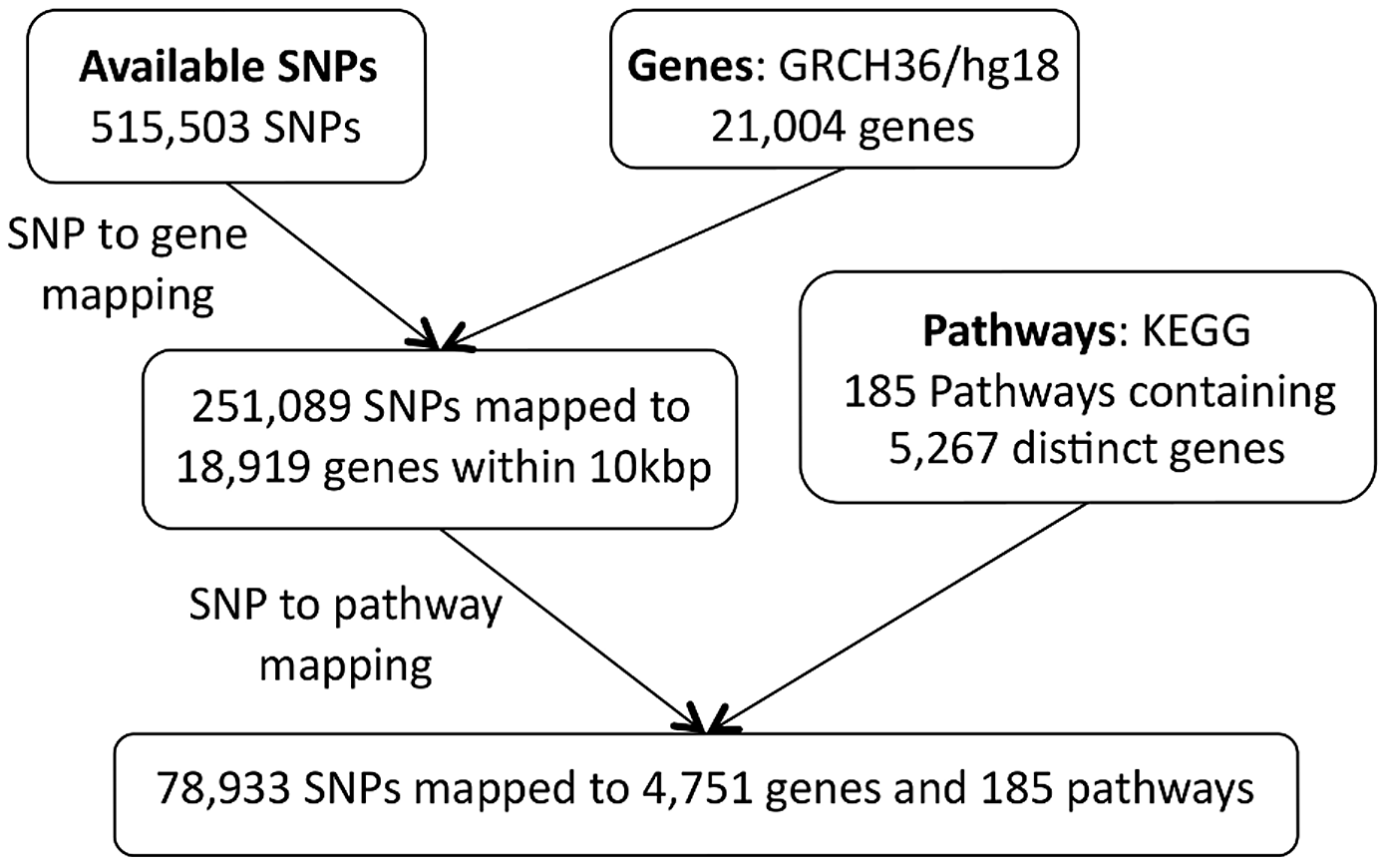
SiMES dataset: SNP to pathway mapping.

Following imputation, SNPs for both datasets in the present study are mapped to KEGG canonical pathways from the MSigDB database (http://www.broadinstitute.org/gsea/msigdb/index.jsp). SNPs are mapped to all genes 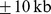, upstream or downstream of the SNP in question. We exclude the largest KEGG pathway (by number of mapped SNPs), ‘Pathways in Cancer’, since it is highly redundant in that it contains multiple other pathways as subsets. Details of the pathway mapping process are given in Figures 9 and 10.

Note that there is a difference in the number of SNPs available for the pathway mapping between the two datasets, and this results in a small discrepancy in the total number of mapped genes (SP2: 4,734 mapped genes; SiMES: 4,751). However, both datasets map to all 185 KEGG pathways, and a large majority of mapped genes and SNPs overlap both datasets. Detailed information on the pathway mapping process for the two datasets is presented in Table 4.

**Table 4:** Comparison of SNP and gene to pathway mappings for the SP2 and SiMES datasets.

	SP2	SiMES
Total SNPs mapping to pathways	75,389	78,933
Total SNPs mapping to pathways in both datasets (intersection)	74,864	
Total mapped genes	4,734	4,751
Total genes mapping to pathways in both datasets (intersection)	4,726	
Total mapped pathways	185	185
Minimum number of genes mapping to single pathway	11	11
Maximum number of genes mapping to single pathway	63	63
Minimum number of SNPs mapping to single pathway	66	67
Maximum number of SNPs mapping to single pathway	5,759	6,058
Minimum number of pathways mapping to a single SNP	1	1
Maximum number of pathways mapping to a single SNP	45	45

### Ethics statement

An ethics statement covering the SP2 and SiMES datasets used in this study can be found in [52].

## Results

We perform pathways-driven SNP selection on the SP2 and SiMES datasets independently using SGL, and combine this with the subsampling procedure described previously to highlight pathways and genes associated with variation in HDLC levels. We present results for each dataset separately, followed by a comparison of the results from both datasets.

### SP2 analysis

For the SP2 dataset we consider two separate scenarios for the regularisation parameters 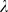 and 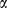. For the two scenarios we set the sparsity parameter, 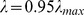, but consider two values for 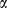, namely 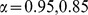. We test each scenario over 1000 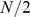 subsamples. We also compare the resulting pathway and SNP selection frequency distributions with null distributions, again over 1000 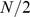 subsamples, but with phenotype labels permuted, so that no SNPs can influence the phenotype.

The parameter 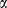 controls how the regularisation penalty is distributed between the 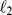 (pathway) and 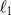 (SNP) norms of the coefficient vector. Each scenario therefore entails different numbers of selected pathways and SNPs, and this information is presented in Table 5.

**Table 5:** Separate combinations of regularisation parameters, 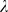 and 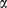 used for analysis of the SP2 dataset.

	*λ* = 0.95*λ_max_*	
	α = 0.85	α = 0.95
*empirical*		
selected pathways	7.9±6.1	4.8±4.1
selected SNPs	1551±1294	160±185
*null*		
selected pathways	9.1±7.2	5.0±4.55
selected SNPs	1656±1401	155±194

For each 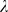, 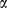 combination, the mean (±SD) number of selected pathways and SNPs across all 1000 subsamples is reported.

**Figure 11:**
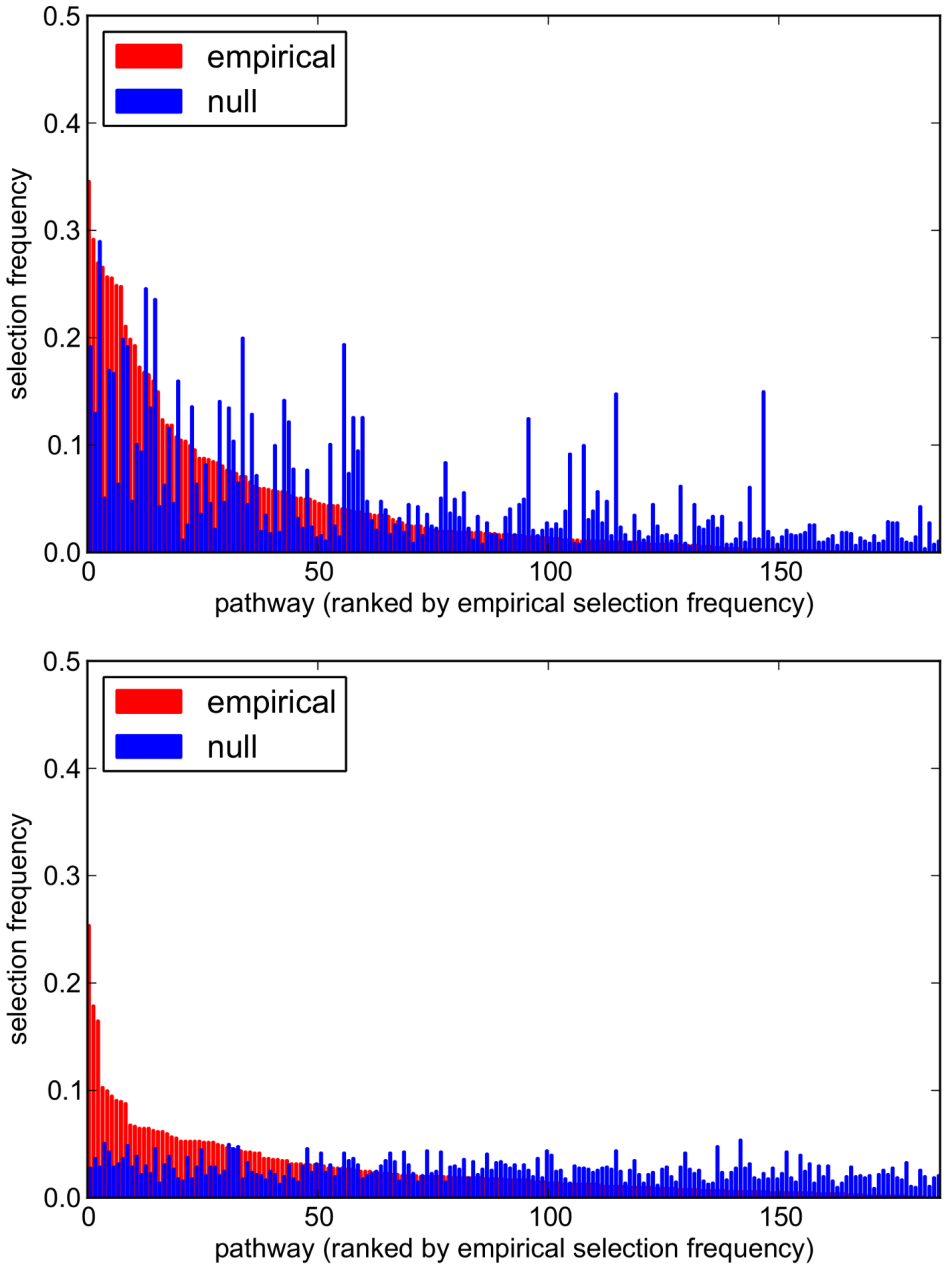
Empirical and null pathway selection frequency distributions for all 185 KEGG pathways with the SP2 dataset. For each scenario, pathways are ranked along the *x*-axis in order of their empirical pathway selection frequency, 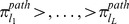. *Top:* 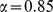. *Bottom:* 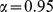.

**Figure 12:**
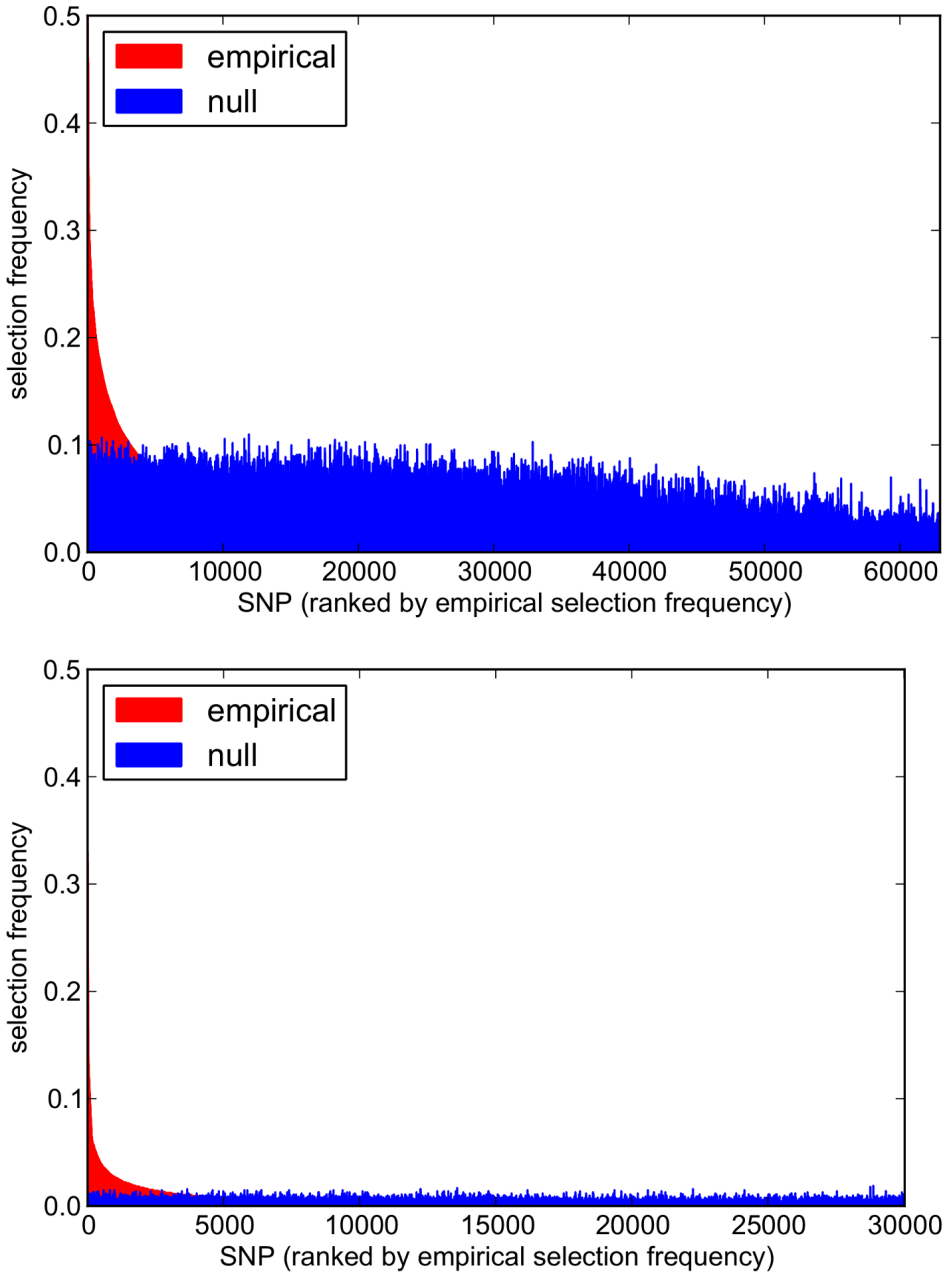
Empirical and null SNP selection frequency distributions with the SP2 dataset. For each scenario, SNPs are ranked along the *x*-axis in order of their empirical pathway selection frequency, 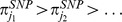. *Top:* 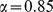. *Bottom:* 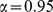. Note fewer SNPs are selected with nonzero empirical selection frequency with 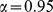, so that the *x*-axis range in the bottom plot is reduced.

Comparisons of empirical and null pathway selection frequency distributions for each scenario are presented in Figure 11. The same comparisons for SNP selection frequencies are presented in Figure 12. In these plots, null distributions (coloured blue) are ordered along the *x*-axis according to their corresponding ranked empirical selection frequencies (marked in red). This is to help visualise any potential biases that may be influencing variable selection.

To interpret these results, we begin by noting from Table 5 that many more SNPs are selected with 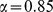, resulting in higher SNP selection frequencies, compared to those obtained with 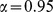 (see Figure 12). This is as expected, since a lower value for 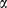 implies a reduced 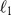 penalty on the SNP coefficient vector, resulting in more SNPs being selected. Perhaps surprisingly, given that the 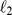 group penalty 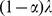 is increased, the number of selected pathways is also greater. This must reflect the reduced 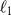 penalty, which allows a greater number of SNPs to contribute to a putative selected pathway's coefficient vector. This in turn increases the number of pathways that pass the threshold for selection.

**Figure 13:**
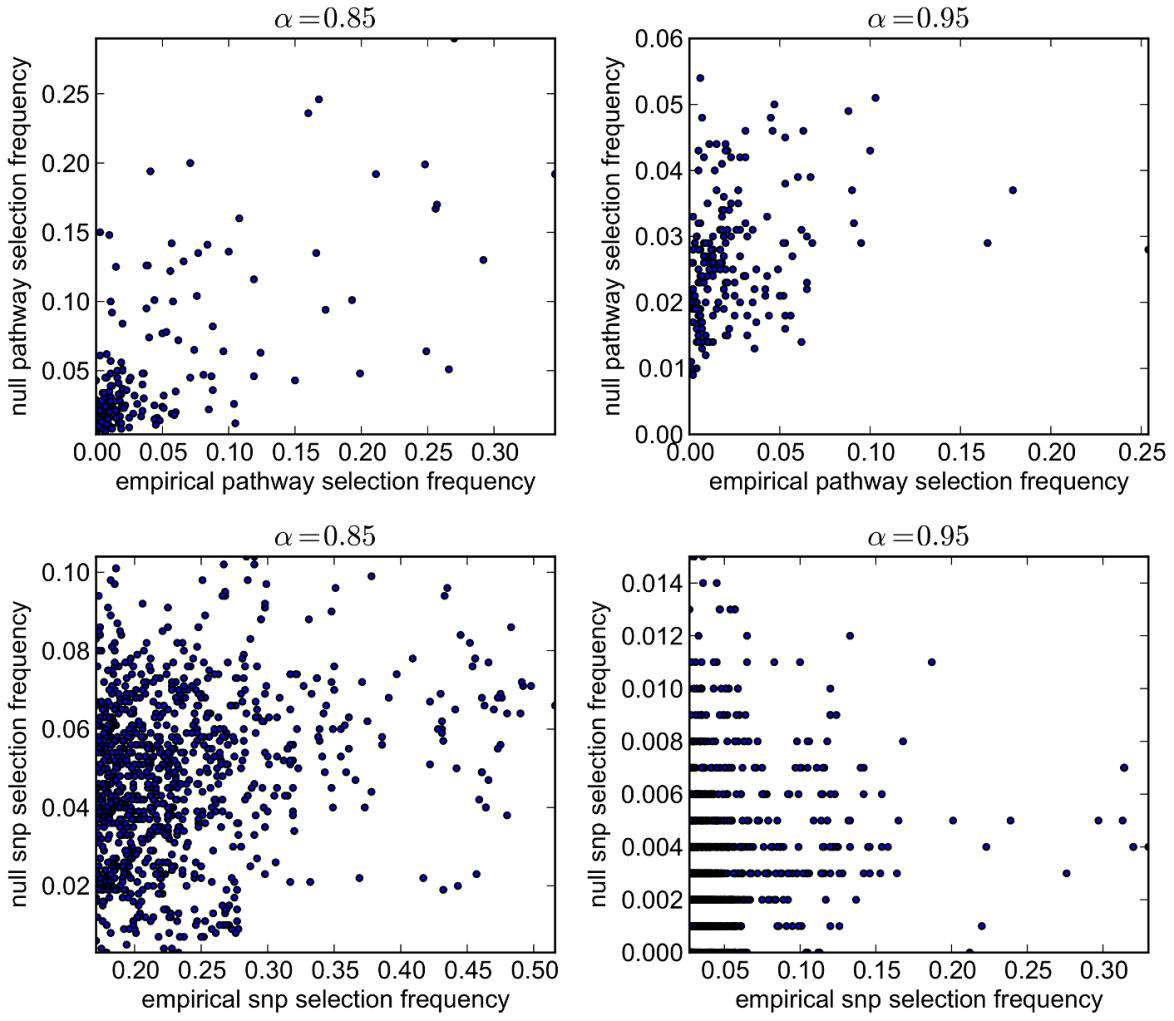
SP2 dataset: scatter plots comparing empirical and null selection frequencies presented in Figures 11 and 12. *Top row:* Pathway selection frequencies with 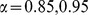. *Bottom row:* SNP selection frequencies for the same 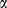 values. For clarity, SNP selection frequencies are plotted for the top 1000 SNPs (by empirical selection frequency) only. Corresponding correlation coefficients (for all ranked SNPs) are presented in Table 6. Note that pathway and SNP selection frequencies are much higher at the lower 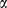 value (left hand plots), since many more variables are selected (see Table 5.)

This raises the question of what might be considered to be an optimal choice for the regularisation-distributional parameter 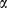, since different assumptions about the number of SNPs potentially influencing the phenotype may affect the resulting pathway and SNP rankings. To answer this, we turn our attention to the pathway and SNP selection frequency distributions for each 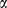 value in Figures 11 and 12. At the lower value of 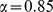 (top plots in Figures 11 and 12), empirical pathway and SNP selection frequency distributions appear to be biased, in the sense that there is a suggestion that pathways and SNPs with the highest empirical selection frequencies also tend to be selected with a higher frequency under the null, where there is no association between genotype and phenotype. This relationship appears to be diminished with 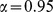, when fewer SNPs are selected by the model. We investigate this further by plotting empirical vs. null selection frequencies as a sequence of scatter plots in Figure 13, and we report Pearson correlation coefficients and p-values for these in Table 6.

**Table 6:** SP2 dataset: Pearson correlation coefficients (*r*) and p-values for the data plotted in Figure 13.

	*α* = 0.85			*α* = 0.95		
	*n*	*r*	p-value	*n*	*r*	p-value
pathways	185	0.66	1.3×10^−24^	185	0.26	2.9×10^−4^
SNPs	62,965	0.37	0	30,027	0.11	1.2×10^−84^

*n* denotes the number of predictors considered. For SNPs, coefficients describe correlations for all predictors selected with nonzero empirical selection frequencies only, since a large number of SNPs are not selected by the model at any subsample.

These provide further evidence of increased correlation between empirical and null selection frequency distributions at the lower 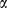 value for both pathways and SNPs, again suggesting increased bias in the empirical results, in the sense that certain pathways and SNPs tend to be selected with a higher frequency, irrespective of whether or not a true signal may be present. Further qualitative evidence of reduced bias with 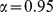 is suggested by the clearer separation of empirical and null distributions at the higher 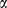 value in Figures 11 and 12. For example, the maximum empirical pathway selection frequency is reduced by a factor of 0.29 (0.35 to 0.25) as 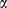 is increased from 0.85 to 0.95, whereas the maximum pathway selection frequency under the null is reduced by a factor of 0.81 (0.29 to 0.054). Similarly for SNPs, the maximum empirical SNP selection frequency is reduced by a factor of 0.37 (0.52 to 0.33), whereas the maximum SNP selection frequency under the null is reduced by a factor of 0.9 (0.11 to 0.011).

The increased bias with 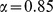 is most likely due to the selection of too many SNPs, in the sense that many selected SNPs do not exhibit real phenotypic effects. These extra SNPs effectively add noise to the model, in the form of multiple weak, spurious signals. This in turn will add bias to the resulting selection frequency distributions, tending to favour, for example, SNPs that overlap multiple pathways, and the pathways that contain them. As 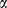 is increased, we would expect this biasing effect to be reduced, until a point where too few SNPs are selected, when there is then a risk that some of the true signal may be lost.

Note that the reduced but still significant correlations between empirical and null selection frequency distributions at 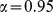 in Table 6 are not unexpected. These may reflect the complex overlap structure between pathways, meaning that pathways (and associated SNPs) with a relatively high degree of overlap with other pathways, due for example to the presence of so called ‘hub genes’, are more likely to harbour true signals, as well as spurious ones [38], [60], [61]. Another potential source of correlations between empirical and null distributions is the effect of LD depressing SNP selection frequencies, highlighted earlier.

Taking all the above into consideration, we choose to report results with 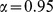, where there is less evidence of bias due to the selection of too many SNPs. The top 30 pathways, ranked by their selection frequency, 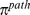 are presented in Table 7, and the top 30 ranked genes, ranked by 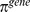 are presented in the left hand part of Table 8. Versions of these tables extending to lower ranks are provided in Tables S1 and S2.

**Table 7:** SP2 dataset: Top 30 pathways, ranked by pathway selection frequency, π*^path^*.

Rank	KEGG pathway name	*π^path^*	Size (# SNPs)	top 30 ranked genes in pathway
1	Toll Like Receptor Signaling Pathway	0.254	766	*TIRAP RAC1 IFNAR1 CD80 IL12B PIK3R1*
2	Jak Stat Signaling Pathway	0.179	1447	*PIAS2 IL5RA TPO IFNAR1 IL12B PIK3R1 IL2RA*
3	Ubiquitin Mediated Proteolysis	0.165	1603	*PIAS2 RFWD2 PARK2*
4	^*^Dilated Cardiomyopathy	0.103	3054	*ADCY2 TGFB3 PRKACB RYR2 ITGB8 ITGA1 CACNA2D3 LAMA2 CACNA1C*
5	Cytokine Cytokine Receptor Interaction	0.100	2553	*IL5RA IL12B TGFB3 EGFR TPO IFNAR1 IL2RA*
6	Ecm Receptor Interaction	0.095	2271	*ITGB8 ITGA1 LAMA2*
7	Arginine And Proline Metabolism	0.091	432	*NOS1*
8	Parkinson's Disease	0.090	1320	*PARK2*
9	^*^ Hypertrophic Cardiomyopathy	0.088	2819	*TGFB3 RYR2 ITGB8 ITGA1 CACNA2D3 LAMA2 CACNA1C*
10	Small Cell Lung Cancer	0.068	1808	*PIAS2 PIK3R1 LAMA2*
11	Natural Killer Cell Mediated Cytotoxicity	0.067	1781	*KRAS RAC1 VAV3 VAV2 PRKCA IFNAR1 PRKCB PIK3R1*
12	^*^ T Cell Receptor Signaling Pathway	0.065	1541	*KRAS VAV3 VAV2 PIK3R1*
13	Tgf Beta Signaling Pathway	0.065	947	*TGFB3*
14	Olfactory Transduction	0.065	2497	*PRKACB*
15	^*^ Arrhythmogenic Right Ventricular Cardiomyopathy	0.063	3726	*RYR2 TCF7L1 ITGB8 ITGA1 CACNA2D3 LAMA2 CACNA1C*
16	^*^ Ppar Signaling Pathway	0.062	758	
17	Taste Transduction	0.062	941	*PRKACB*
18	Type I Diabetes Mellitus	0.060	776	*CD80 IL12B*
19	^*^ Ribosome	0.057	261	
20	^*^ Terpenoid Backbone Biosynthesis	0.056	147	
21	Neuroactive Ligand Receptor Interaction	0.053	5745	*GRIN3A*
22	Regulation Of Actin Cytoskeleton	0.053	3803	*KRAS RAC1 EGFR ITGB8 VAV3 ITGA1 VAV2 PIK3R1*
23	Mismatch Repair	0.053	222	
24	Cell Adhesion Molecules Cams	0.053	3977	*ITGB8 CD80*
25	Maturity Onset Diabetes Of The Young	0.053	239	
26	Butanoate Metabolism	0.052	383	
27	Purine Metabolism	0.052	3224	*ADCY2*
28	P53 Signaling Pathway	0.052	598	*RFWD2*
29	Dorso Ventral Axis Formation	0.050	581	*KRAS EGFR*
30	Basal Cell Carcinoma	0.049	589	*TCF7L1*

The final column lists genes in the pathway that are in the top 30 ranked genes selected in the study (see left-hand side of Table 8). Pathways falling in the consensus set, 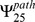, obtained by comparing pathway ranking results from both SP2 and SiMES datasets (see Table 11), are marked with a 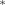.

**Table 8:** SP2 and SiMES datasets: Top 30 genes ranked by gene selection frequency, 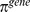.

	SP2 GENE RANKING			SiMES GENE RANKING		
Rank	Gene	*π^gene^*	# mapped SNPs	Gene	*π^gene^*	# mapped SNPs
1	*IFNAR1*	0.33	11	*PPA2*	0.31	16
2	*IL12B*	0.3	9	*PDSS2*	0.26	59
3	*PIAS2*	0.3	7	*GABARAPL1*	0.18	11
4	*TIRAP*	0.22	5	*ATP6V0A4*	0.15	35
5	*RAC1*	0.21	10	*ITGB1*	0.13	14
6	*LAMA2* ^*^	0.19	111	*CACNA1C* ^*^	0.11	186
7	*ADCY2* ^*^	0.19	94	*PRKCB* ^*^	0.11	84
8	*PIK3R1*	0.19	28	*FYN*	0.11	46
9	*PARK2*	0.19	460	*BCL2* ^*^	0.1	61
10	*IL2RA*	0.19	55	*PAK7* ^*^	0.1	127
11	*PRKCA* ^*^	0.19	123	*DGKB*	0.1	233
12	*ITGB8*	0.18	27	*LAMA2* ^*^	0.1	118
13	*TCF7L1*	0.18	55	*NDUFA4*	0.1	7
14	*CD80* ^*^	0.18	21	*DGKH*	0.1	70
15	*GRIN3A*	0.18	60	*ADCY2* ^*^	0.09	104
16	*PRKCB* ^*^	0.18	83	*LIPC*	0.09	69
17	*CACNA1C* ^*^	0.17	180	*SLC8A1* ^*^	0.09	240
18	*TGFB3*	0.16	7	*EGFR* ^*^	0.09	74
19	*PRKACB*	0.16	16	*PRKAG2*	0.09	118
20	*KRAS* ^*^	0.16	21	*CACNA1D*	0.09	83
21	*VAV3*	0.16	97	*ITGA11* ^*^	0.09	63
22	*IL5RA*	0.15	38	*IGF1R* ^*^	0.09	100
23	*ITGA1* ^*^	0.15	77	*SDHC*	0.09	9
24	*VAV2* ^*^	0.15	85	*CACNA2D3* ^*^	0.08	294
25	*EGFR* ^*^	0.14	61	*RYR2* ^*^	0.08	221
26	*TPO*	0.14	50	*ITGA1* ^*^	0.08	77
27	*CACNA2D3* ^*^	0.14	283	*ALDH7A1*	0.08	23
28	*RYR2* ^*^	0.14	214	*MGST3* ^*^	0.08	40
29	*NOS1*	0.14	49	*ALDH2*	0.08	12
30	*RFWD2*	0.13	31	*SDHB*	0.08	13

Genes falling in the top 30 ranks of the consensus gene set, π_244_^*gene*^, obtained by comparing gene ranking results from both SP2 and SiMES datasets (see Table 13), are marked with a *.

### SiMES analysis

**Figure 14:**
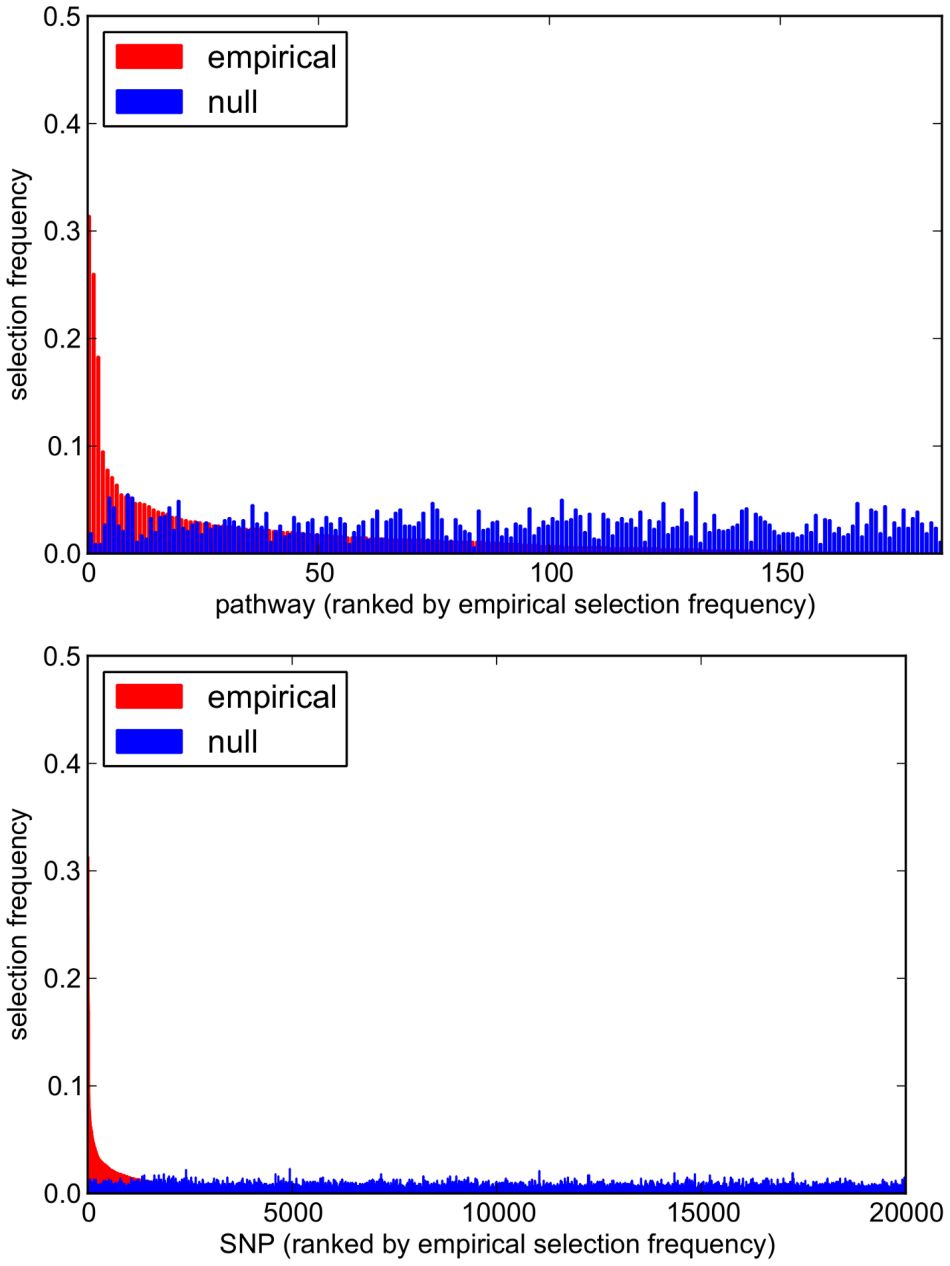
Empirical and null pathway (*top*) and SNP (*bottom*) selection frequency distributions for the SiMES dataset. 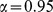
. For both empirical (red) and null (blue) distributions, variables (pathways and SNPs) are ranked along the *x*-axis in order of their empirical selection frequencies.

**Figure 15:**
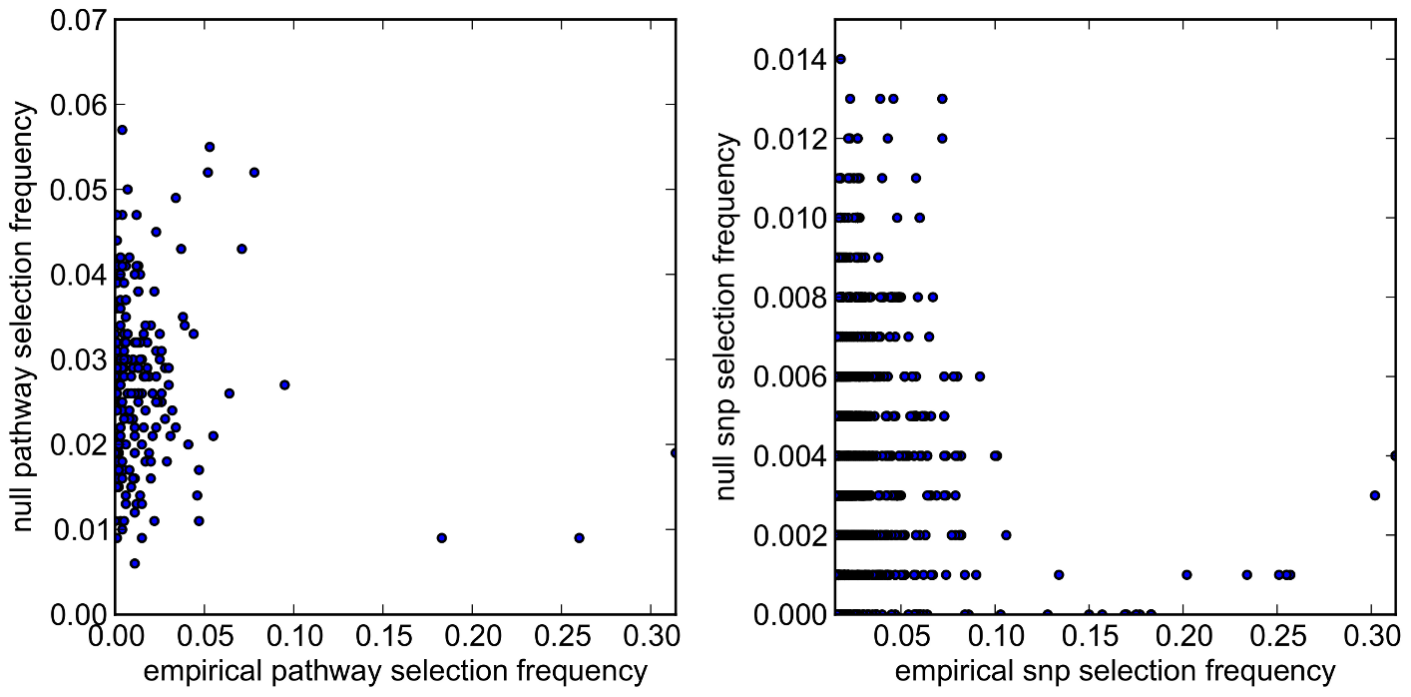
SiMES dataset: Scatter plots comparing empirical and null pathway (*left*) and SNP (*right*) selection frequencies presented in Figure 14. For clarity, SNP selection frequencies are plotted for the top 1000 SNPs (by empirical selection frequency) only.

For the replication SiMES dataset, we repeat the above analysis design, but consider only the ‘low bias’ scenario where 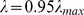 and 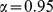. Once again we test each scenario over 1000 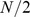 subsamples, and compare the resulting pathway and SNP selection frequency distributions with null distributions generated over 1000 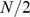 subsamples with phenotype labels permuted. Pathway and SNP selection frequency distributions are presented in Figure 14. An investigation of pathway and SNP selection bias is presented in the form of scatter plots illustrating potential correlation between empirical and null selection frequencies in Figure 15, with corresponding Pearson correlation coefficients and p-values presented in Table 9. The top 30 ranked pathways and genes are presented in Tables 10 and 8 (right hand part) respectively, and extended rankings are provided in Tables S3 and S4.

**Table 9:** SiMES dataset: Pearson correlation coefficients (*r*) and p-values for the data plotted in Figure 15.

	*n*	*r*	p-value
pathways	185	−0.094	0.20
SNPs	20,006	0.058	2.63×10^−6^

Refer to Table 6 for details.

**Table 10:** SiMES dataset: Top 30 pathways, ranked by pathway selection frequency, *π^path^*.

Rank	KEGG pathway name	*π^path^*	Size (# SNPs)	top 30 ranked genes in pathway
1	Oxidative Phosphorylation	0.314	871	*PPA2 NDUFA4 SDHB SDHC ATP6V0A4*
2	^*^ Terpenoid Backbone Biosynthesis	0.260	158	*PDSS2*
3	Regulation Of Autophagy	0.183	215	*GABARAPL1*
4	Glycerolipid Metabolism	0.095	1074	*ALDH7A1 DGKB DGKH ALDH2 LIPC*
5	^*^ Dilated Cardiomyopathy	0.078	3177	*ADCY2 RYR2 ITGA11 ITGB1 SLC8A1 ITGA1 CACNA2D3 LAMA2 CACNA1C CACNA1D*
6	^*^ Hypertrophic Cardiomyopathy	0.071	2932	*PRKAG2 RYR2 ITGA11 ITGB1 SLC8A1 ITGA1 CACNA2D3 LAMA2 CACNA1C CACNA1D*
7	^*^ Ribosome	0.064	270	
8	Glutathione Metabolism	0.055	389	*MGST3*
9	^*^ Arrhythmogenic Right Ventricular Cardiomyopathy	0.053	3899	*RYR2 ITGA11 ITGB1 SLC8A1 ITGA1 CACNA2D3 LAMA2 CACNA1C CACNA1D*
10	^*^ T Cell Receptor Signaling Pathway	0.052	1624	*PAK7 FYN*
11	Cardiac Muscle Contraction	0.047	1952	*RYR2 SLC8A1 CACNA2D3 CACNA1C CACNA1D*
12	Biosynthesis Of Unsaturated Fatty Acids	0.047	282	
13	Lysosome	0.046	1322	*ATP6V0A4*
14	Apoptosis	0.044	954	*BCL2*
15	Pathogenic Escherichia Coli Infection	0.041	538	*ITGB1 FYN*
16	Metabolism Of Xenobiotics By Cytochrome P450	0.039	880	*MGST3*
17	Drug Metabolism Cytochrome P450	0.038	910	*MGST3*
18	Autoimmune Thyroid Disease	0.037	686	
19	Focal Adhesion	0.034	4787	*ITGA11 LAMA2 BCL2 FYN EGFR ITGB1 ITGA1 PAK7 PRKCB IGF1R*
20	Leishmania Infection	0.034	718	*PRKCB ITGB1*
21	^*^ Ppar Signaling Pathway	0.032	800	
22	Rna Polymerase	0.031	193	
23	Lysine Degradation	0.030	423	*ALDH7A1 ALDH2*
24	Endocytosis	0.030	3436	*EGFR IGF1R*
25	Glycosaminoglycan Biosynthesis Chondroitin Sulfate	0.029	727	
26	Melanoma	0.028	1189	*EGFR IGF1R*
27	Nucleotide Excision Repair	0.028	330	
28	Prostate Cancer	0.026	1419	*EGFR IGF1R BCL2*
29	Renal Cell Carcinoma	0.026	1004	*PAK7*
30	Glycine Serine And Threonine Metabolism	0.026	268	

The final column lists genes in the pathway that are in the top 30 ranked genes selected in the study (i.e. genes in the top 30 gene rankings in the right-hand side of Table 8). Pathways falling in the consensus set, 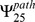, obtained by comparing pathway ranking results from both SP2 and SiMES datasets (see Table 11), are marked with a ^*^.

### Comparison of ranked pathway and gene lists

We now consider the problem of comparing the pathway and gene rankings obtained for each dataset. To do this we require some measure of distance between each pair of ranked lists. Ideally this measure should place more emphasis on differences between highly-ranked variables, since we expect the association signal, and hence agreement between the ranked lists, to be strongest there. By the same reasoning, we expect there to be little or no agreement between variables at lower rankings, where selection frequencies are low. Indeed a consideration of empirical and null selection frequency distributions (Figures 11 (bottom), 12 (bottom) and 14) suggests that only the very top ranked variables are likely to reflect any true signal, so that we would additionally like our distance metric to be able to accommodate consideration of the top-*k* variables only, with 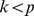, where *p* is the total number of variables ranked in either dataset. One complication with top-*k* lists is that they are *partial*, in the sense that unlike complete 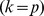 lists, a variable may occur in one list, but not the other.

In order to consider this problem, we introduce the following notation. We denote the complete set of ranked predictors by 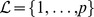, and begin by assuming that all variables are ranked in both datasets. We denote the rank of each variable in list 1 by 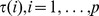, so that 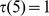 if variable 5 is ranked first and so on. The corresponding ranks for list 2 are denoted by 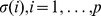. A suitable metric describing the distance between two top-*k* rankings is the *Canberra distance* [62],
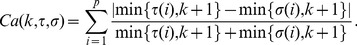
(6)This has the properties that we require, in that the denominator ensures more emphasis is placed on differences in the ranks of highly ranked variables in either dataset. Furthermore, this distance measure allows comparisons between partial, top-*k* lists, since a variable occurring in one top-*k* list but not the other is assigned a ranking of 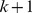 in the list from which it is missing. Note also that a variable *i* that is not in either of the top-*k* ranks, that is 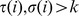, makes no contribution to 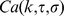.

In order to gauge the extent to which the distance measure (6) differs from that expected between two random lists, we require a value for the expected Canberra distance between two random lists, which we denote 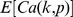. Jurman et al. [62] derive an expression for this quantity, and we use this to compute the normalised Canberra distance,
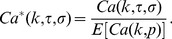
(7)


Note that this has a lower bound of 0, corresponding to exact agreement between the lists. For two random lists, the upper bound will generally be close to 1, although it can exceed 1, particularly for small *k*, since the expected value for random lists is not necessarily the highest value.

#### Pathway rankings

**Figure 16:**
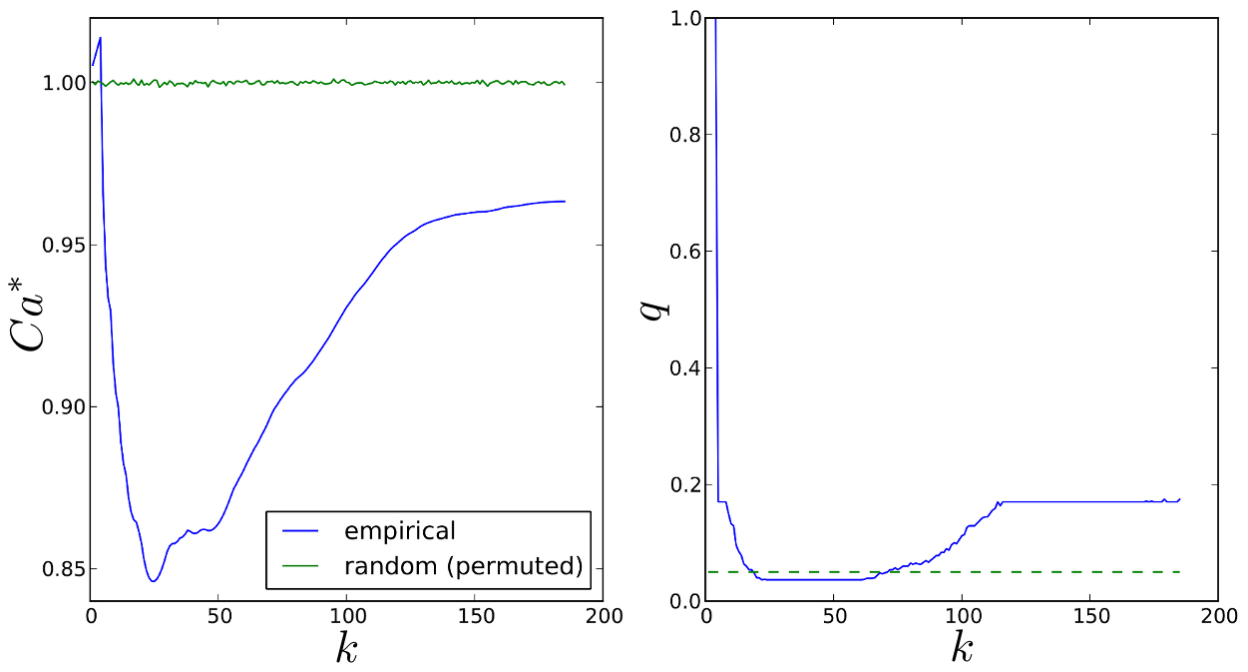
Comparison of top-*k* SP2 and SiMES pathway rankings. *Left:* Variation of normalised Canberra distance, 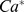 with *k* (7) (blue curve). Corresponding mean values over 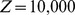 permutations of SiMES rankings (8) (green curve). *Right:* FDR *q*-values (blue curve). Dotted green line shows the threshold for FDR control at the 5% level.

We illustrate the variation of the normalised Canberra distance (7) between SP2 and SiMES pathway rankings in the left hand plot in Figure 16 (blue curve). We consider all possible top-*k* lists, 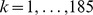 since all 185 pathways are ranked in both datasets. In the same plot, we also show
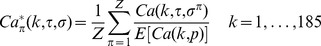
(8)obtained by comparing empirical SP2 rankings 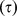 against 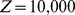 permutations of the SiMES pathway rankings, 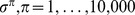 (green curve). This latter curve confirms that the expected value, 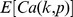, is indeed a good measure of 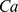 in the random case where there is no agreement between rankings.

Using the same permuted rankings, 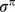, we next test the null hypothesis that the observed normalised Canberra distance, 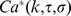, is not significantly different from that between 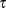 and a random list 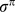, by computing a p-value as
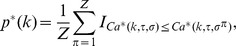
for 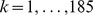. We then obtain FDR q-values using the Benjamini-Hochberg procedure [63] and illustrate these for each *k* in the right hand plot of Figure 16. FDR is controlled at a nominal 5% level for 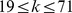, indicating that the distance between the top-*k* pathway rankings for both datasets is significantly different from the random ranking case for a wide range of possible values of *k*. The distance 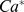 between SP2 and SiMES pathway rankings however attains its minimum value when 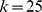 with q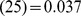, so that on this measure, the two pathway rankings are in closest agreement when we consider the top 25 pathways in each ranked list only. Some intuitive understanding of why this might be so can be gained by considering the empirical vs. null pathway selection frequency distributions for each dataset in Figures 11 (bottom) and 14 (top). Here we see that the separation between empirical and null selection frequencies is most clear for values of *k* below around 30 for SP2, and around 15 for SiMES.

If we assume that the two pathway rankings are indeed in closest agreement when 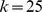, then one means of obtaining a consensus set of important pathways is to consider their intersection,
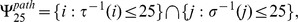
from which we can obtain a set of average rankings as
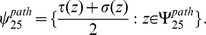
Both the intersection set, 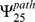, and ordered average rankings, 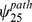 for the two datasets under consideration are shown in Table 11. We additionally mark the consensus set 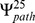 with asterisks in Tables 7 and 10.

**Table 11:** Consensus set of pathways, 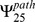, for SP2 and SiMES datasets with *k* = 25.

Pathway	Average rank ( 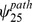 )
Dilated Cardiomyopathy	4.5
Hypertrophic Cardiomyopathy	7.5
T Cell Receptor Signaling Pathway	11.0
Terpenoid Backbone Biosynthesis	11.0
Arrhythmogenic Right Ventricular Cardiomyopathy	12.0
Ribosome	13.0
Ppar Signaling Pathway	18.5

Consensus pathways are ordered by their average rankings in 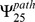.

#### Gene rankings

A number of factors complicate the comparison of ranked gene lists across both datasets. Firstly, sets of mapped genes differ slightly between the two datasets (see Table 3). Secondly, even if we consider only those variables *mapped* in both datasets, different, though overlapping sets of variables are *ranked* in each. Thirdly, ranked variables are not independent [62]. For example, genes may be grouped into pathways, so that a reordering of genes within a pathway might be considered less significant than a reordering of genes mapping to different pathways.

In order to compute a distance measure between pairs or ranked gene lists, we therefore make two simplifying assumptions. First, we consider only genes ranked in one or both datasets. This seems reasonable, since we can necessarily only compile a distance measure from variables that are ranked in one or both datasets. Second, we assume that genes are independent. This makes our distance measure conservative, in the sense that it will treat all reordering of genes equally, irrespective of any potential functional relationship between them.

With these assumptions in mind, we begin by denoting the set of all 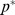 genes that are ranked in *either* dataset by 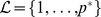. We further denote the corresponding sets of ranked genes for SP2 and SiMES datasets by 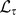 and 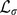 respectively. We then have the following set relations: 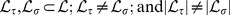.

We now extend the previous Canberra distance measure to encompass the above set relations. We begin, as before, by defining two ranked lists corresponding to gene rankings in 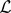 for each dataset, although this time we must account for the fact that not all variables in 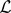 are ranked in both. We denote SP2 rankings by 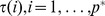, where 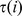 is the rank of gene *i* if 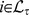, and 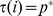 otherwise. SiMES rankings are defined in the same way, and denoted by 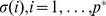.

Applying this revised ranking scheme, we can then define a top-*k* normalised Canberra distance (6) as
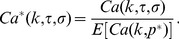
(9)for any 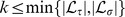. The restriction on *k* follows from the fact that we cannot distinguish between top-*k* rankings for all 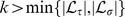.

**Figure 17:**
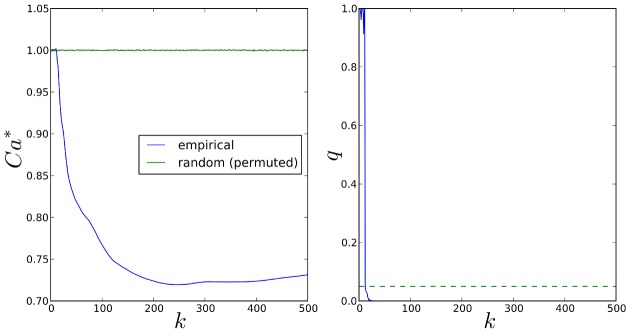
Comparison of top-*k* SP2 and SiMES gene rankings, for 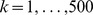
 **.** *Left:* Variation of normalised Canberra distance, 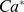 with *k* (9) (blue curve), and corresponding mean values over 10,000 permutations of SiMES rankings (8) (green curve). *Right:* FDR *q*-values (blue curve). Dotted green line shows the threshold for FDR control at the 5% level.

Information summarising the relationship between the two ranked lists of genes is given in Table 12. We consider normalised Canberra distances, 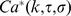, for 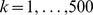 only, and plot these in Figure 17 (left, blue curve), along with 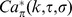 (8) for 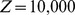 permutations of the SiMES gene rankings, 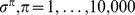 (green curve). Once again this latter curve confirms that the expected value, 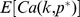, is indeed a good measure of *Ca* in the random case where there is no agreement between rankings. We also plot FDR *q*-values using the same procedure as described previously for pathways. FDR is controlled at a nominal 5% level for all 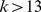 in the region tested 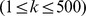. The distance 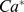 between SP2 and SiMES gene rankings attains its minimum value when 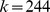, so that on this measure, the two gene rankings are in closest agreement when we consider the top 244 genes in each ranked list only.

**Table 12:** Summary of genes analysed and ranked in SP2 and SiMES datasets.

	SP2	SiMES
number of genes mapped to pathways	4,734	4,751
number of genes mapping to both datasets	4,726	
number of ranked genes ( 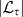 , 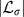 )	3,430	2,815
number of genes ranked in either dataset (*p* ^*^)	3,913	
number of genes ranked in both datasets ( 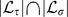 )	2,332	

Following the same strategy as implemented for pathways, we then form the consensus set, 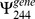, and average rankings 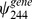. The consensus set contains 84 genes, and we list the top 30 genes ordered by their average rank in the two datasets, in Table 13.

**Table 13:** Top 30 consensus genes ordered by their average rank, 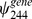.

Rank	Gene	Average rank ( 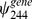 )
1	*LAMA2*	9.0
2	*ADCY2*	11.0
3	*CACNA1C*	11.5
4	*PRKCB*	11.5
5	*PRKCA*	21.0
6	*EGFR*	21.5
7	*ITGA1*	24.5
8	*CACNA2D3*	25.5
9	*RYR2*	26.5
10	*IGF1R*	30.5
11	*PAK7*	36.5
12	*ADCY8*	37.5
13	*VAV2*	41.0
14	*SLC8A1*	41.5
15	*CACNB2*	42.5
16	*CACNA2D1*	43.0
17	*ITGA9*	44.0
18	*KRAS*	47.5
19	*MAPK10*	50.5
20	*CACNA1S*	51.0
21	*VAV3*	54.0
22	*PLCG2*	55.5
23	*BCL2*	57.0
24	*CD80*	60.0
25	*ITGA11*	60.5
26	*CTNNA2*	61.0
27	*ALDH1B1*	61.5
28	*MGST3*	63.0
29	*NEDD4L*	63.0
30	*PRKAG2*	66.0

### Comparisons with SNP GWAS

Finally, we compare gene rankings for each cohort obtained using our method with those from a standard GWAS in which SNPs are tested separately for their association with HDLC. Results from the latter study form part of an ongoing multi-cohort study and so are reported in summary form only. Further details are presented in Supplementary Information S1, Section 6. By considering only SNPs that map to pathways in each cohort, we find that the top 50 ranked genes using our method are highly enriched amongst genes mapping to highly-ranked SNPs in their respective GWAS (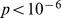 by permutation). Furthermore 4 out of the top 10 ranked genes in the SP2 dataset using our method are also in the top 10 of 4,734 genes ranked in the SP2 GWAS. The corresponding figure for the SiMES cohort is 2 out of 10. As with our gene ranking results (Table 8), we find little concordance between high ranking genes in both GWAS, with for example no gene occurring amongst the top 10 gene ranks in both cohorts. Note that none of the subset of SNPs in either GWAS that map to pathways in our study achieves genome-wide significance after correcting for multiple testing (SP2 cohort, 75,389 SNPs, minimum SNP p-value = 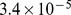; SiMES cohort, 78,933 SNPs, minimum SNP p-value = 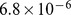).

## Discussion

We have outlined a method for the detection of pathways and genes associated with a quantitative trait. Our method uses a sparse regression model, the sparse group lasso, that enforces sparsity at the pathway and SNP level. As well as identifying important pathways, this model is designed to maximise the power to detect causal SNPs, possibly of low effect size, that might otherwise be missed if pathways information is ignored. In a simulation study we demonstrated that where causal SNPs are enriched within a single causal pathway, SGL does indeed have greater SNP selection power, compared to an alternative sparse regression model, the lasso, that disregards pathways information. These results mirror previous findings that support the intuition that a sparse selection penalty that promotes dual-level sparsity is better able to recover the true model in these circumstances [20], [21].

We then argued from a theoretical standpoint that where individual SNPs can map to multiple pathways, a modification (SGL-CGD) of the standard SGL-BCGD estimation algorithm that treats pathways as independent, may offer greater sensitivity for the detection of causal SNPs and pathways. A potential concern is that this gain in power may be accompanied by an inflated number of false positives. However, in a simulation study with overlapping pathways we found relative gains in both sensitivity and specificity under the independence assumption. This gain in specificity was unexpected, and appears to arise directly from treating pathways as independent in the model estimation.

Our method combines the SGL model and SGL-CGD estimation algorithm with a weight-tuning algorithm to reduce selection bias, and a resampling technique designed to provide a robust measure of variable importance in a finite sample. As such, the latter is expected to confer advantages, in terms of the down ranking of unimportant predictors, previously observed for the lasso [45], [47]. As with the group lasso, the ability of SGL to recover the true model is likely to be affected by the complexity of the pathway overlap structure [64], as well as complex patterns of SNP LD. For this reason we test our approach in a final simulation study using real genotype and pathways data. In doing so we confirm previous findings that in the presence of widespread LD, the use of data resampling procedures in combination with a lasso penalty for SNP selection can result in loss of power [49]. However, if we instead measure gene selection frequencies by recording genes mapping to selected SNPs at each subsample, our method shows enhanced power and specificity when compared to a regression-based quantitative trait test that ignores pathways information.

We do not explore the issue of determining a selection frequency threshold for the control of false positives here. In principal such a threshold could be determined by comparing empirical selection frequency distributions with those obtained under the ‘null’ through permutations, although this is not a trivial exercise [65]. An alternative method for error control has been investigated in the context of lasso selection [45], but the direct application of this approach to the present case is not feasible, since overlapping pathways make clear distinctions between causal and noise variables problematic. We instead develop a heuristic measure of ranking performance in our application study identifying genes and pathways associated with serum high-density lipoprotein cholesterol levels (HDLC). Firstly, by comparing empirical and null pathway and SNP rankings for each dataset, we gain some confidence that pathway and SNP signals captured in the top rankings can be distinguished from those arising from noise or spurious associations. Secondly, we take advantage of the fact that we are able to compare results from two independent GWAS datasets. On the assumption that similar patterns of genetic variation are likely to impact HDLC levels in both cohorts, we set a ranking threshold based on computing distances between ranked lists of pathways and genes from each dataset.

Interestingly, when a comparison between empirical and null rankings is made with a reduced value for the regularisation parameter 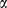, there is evidence of selection bias, in the sense that pathways and SNPs tend to be highly ranked both empirically and under the null. Since a smaller 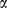 corresponds to a greater number of SNPs being selected at each subsample, this would seem to suggest that too many SNPs are being selected. In this case, pathway and gene rankings (derived from selected SNPs) may in part reflect spurious associations, with a bias towards SNPs overlapping multiple pathways.

Many pathways analysis methods can be categorised as being either competitive or self-contained, according to the type of null hypothesis that is tested [6], [66]. With self-contained or association-type methods, pathway, SNP or gene statistics are tested against the null hypothesis of no association. In contrast, competitive or enrichment-type methods test the null hypothesis that genes or SNPs in a pathway are no more associated with the phenotype than those not in the pathway. Methods testing the self-contained null hypothesis can be more powerful than competitive tests, although at the expense of increased type-I errors, particularly in the context of GWAS data where test statistics may be inflated by stratification or cryptic relatedness [67]. Since our method performs variable selection and does not perform hypothesis testing it cannot strictly be classified as a competitive or association-type method. However, we note that elements of the approach we take in our HDLC application study bear some similarity with competitive-type methods. In particular our use of variable rankings, along with genome-wide comparisons of empirical and ‘null’ (permuted) pathway and SNP selection frequencies guard against genome-wide exaggeration of variables' importance, by comparing variable selection frequencies across all pathways.

There are other potentially interesting areas to explore with regard to the subsampling method used here. For example, standard approaches consider only the set of variables selected at each subsample, and ignore potentially relevant information captured in the coefficient estimates themselves. The use of this additional information would result in a set of ranked lists, one for each subsample, and the joint consideration of these lists has the potential to provide a more robust measure of variable importance, by taking account of the relative importance of each variable for each subsample [68]–[70].

Turning to the study results, we conduct two separate analyses on independent discovery and replication datasets. Since subjects from both datasets are genotyped on the same platform, the large majority of SNPs mapping to pathways in one dataset do so also in the other dataset. Thus 99.3% of SNPs mapping to pathways in the SP2 dataset are similarly mapped in the SiMES dataset. For the SiMES dataset, the corresponding figure is 94.8%. As expected, the concordance of gene coverage is even greater. Thus 99.8% of mapped genes in the SP2 dataset are also mapped in the SiMES dataset, and 99.5% of mapped genes in the SiMES dataset are also mapped in SP2. This large overlap in gene (and pathway) coverage between datasets is likely to occur even when datasets are genotyped on different SNP arrays. Indeed this is one advantage of methods such as the one described here that enable comparisons between pathway and gene rankings.

We obtain consensus pathway and gene rankings by considering only the top *k* ranks in each dataset, with *k* obtained as the value that minimises the distance between the two rankings. We additionally derive a significance measure for each top-*k* distance by comparing empirical distances against a null distribution obtained by permuting ranks in one list. We note that this can only be an approximation of the true null, since in reality rankings for both datasets may be influenced by the extent to which genes and SNPs overlap multiple pathways. However, some support for the reasonableness of this approximation can be gained from our earlier analysis, showing that the correlation between empirical and null pathway and SNP rankings is low, so that rankings under the null are indeed approximately random.

Considering the consensus pathway rankings in Table 11, three out of the seven consensus pathways (ranked 1, 2 and 5), are related to cardiomyopathy. These three pathways are the only cardiomyopathy-related pathways amongst the 185 KEGG pathways used in our analysis, so it is noteworthy that all three fall within the consensus pathway rankings. The link between HDLC levels and cardiomyopathy is already well established [31], [71]–[74]. Furthermore, numerous references in the literature also describe the links between lipid metabolism and T cell receptor (consensus pathway ranking 3) and PPAR signaling (rank 7) [75]–[78].

Turning to a consideration of the top 30 consensus genes presented in Table 13 and (and see also pathway ranking tables 7, 10 and 11, and extended results in Tables S1, S2, S3, S4). We found that many are enriched in one of several gene families:

1. L-type calcium channel genes, including *CACNA1C*, *CACNA1S*, *CACNA2D1*, *CACNA2D3* and *CACNB2*
2. Adenylate cyclase genes, including *ADCY2*, *ADCY4* and *ADCY8*
3. Integrin and laminin genes, including *ITGA1*, *ITGA9*, *ITGA11*, *LAMA2*, and *LAMA3*
4. MAPK signaling pathway genes, including *MAPK10* and *MAP3K7*
5. Immunological pathway genes, including *PAK2*, *PAK7*, *PRKCA*, *PRKCB*, *VAV2* and *VAV3*

These genes are highly enriched in several high ranking pathways from both datasets. Notably, the focal adhesion pathway alone has 12 gene hits, as does the dilated cardiomyopathy pathway. Cardiomyopathy pathways as a whole have 30 genes hits (several of the genes overlap more than one cardiomyopathy pathway). 10 of these genes feature in the MAPK signaling pathway, while GnRH (8 genes), T and B cell receptor (8), calcium (7), ErbB (5), and Wnt signling (4) pathways also contain several genes in the list. To elucidate the biological relevance of these gene families and the connections between them, we investigated their known functional links with cardiovascular phenotypes (not restricted to HDLC) by referencing the KEGG and Genetic Association (http://geneticassociationdb.nih.gov) databases.

### 

#### Voltage dependent L-type calcium channel gene family

The genes in this family encode the subunits of the human voltage dependent L-type calcium channel (CaV1). The 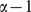 subunit (encoded by *CACNA1C*, *A1S*, *A2D1* and *A2D3* in our study) determines channel function in various tissues. CaV1 function has significant impact on the activity of heart cells and smooth muscles. For example, patients with malfunctioning CaV1 develop arrhythmias and shortened QT interval [79]–[81]. Furthermore, *CACNA1C* polymorphisms have been associated with variation in blood pressure in Caucasian and East Asian populations by pharmacogenetic analysis. In 120 Caucasians, 3 SNPs in this gene were significantly associated with the response to a widely applied antihypertensive CaV1 blocker [82]. Kamide et al. [83] also found that polymorphisms in *CACNA1C* were associated with sensitivity to an antihypertensive in 161 Japanese patients. The CaV1 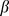 subunit encoding *CACNB2* has also been associated with blood pressure [84].

This gene family was mapped to several pathways in our study, with the KEGG dilated cardiomyopathy pathway achieving highest rank both within individual datasets, and in the consensus pathway rankings. Dilated cardiomyopathy is the most common form of cardiomyopathy, and features enlarged and weakened heart muscles. Although high levels of serum HDLC lowers the risk of heart disease [31], [85], there is still no direct evidence that CaV1 is involved in HDLC metabolism.

#### Adenylate cyclase gene family

Three adenylate cyclases genes, *ADCY2*, *ADCY4* and *ADCY8* were highly ranked in our study. Currently, there are no reported associations of these genes with cardiovascular disease or lipid levels. Adenylate cylcase genes catalyse the formation of cyclic adenosine monophosphate (cAMP) from adenosine triphosphate (ATP), while cAMP servers as the second messenger in cell signal transduction. Note that *ADCY2* is insensitive to calcium concentration, suggesting that any association of this gene family with HDLC levels may not be due to any interactions with the CaV1 gene family.

Among high ranking pathways, *ADCY2* and *ADCY8* feature in the dilated cardiomyopathy pathway.

#### Integrin and laminin gene families

We found 3 genes encoding integrin subunits in our study. Integrins hook to the extracellular matrix (ECM) from the cell surface, and are also important signal transduction receptors which communicate aspects of the cell's physical and chemical environment [86]. Interestingly, laminins are the major component of the ECM, and are relevant to the shape and migration of almost every type of tissue. Both of these two families of genes are therefore highly relevant to the survival and shape of heart muscles. A recent GWAS conducted in a Japanese population confirmed a previous association between *ITGA9* and blood pressure in European populations [87].

Integrin family genes and *LAMA2* were selected primarily within high-ranking cardiomyopathy, focal adhesion and ECM receptor signaling pathways, with once again the dilated cardiomyopathy pathway achieving the highest ranks. However, evidence for *LAMA3* association is weaker, since it was not in the top 30 consensus genes.

#### MAPK signaling pathway

TAK1 (*MAP3K7*) and JNK3 (*MAPK10*) are kinases which regulate cell cycling. They activate or depress downstream transcription factors which mediate cell proliferation, differentiation and inflammation.

JNK activity has been associated with obesity in a mouse model, where the absence of JNK1 (*MAPK8*), a protein in the same family as *MAPK10*, protects against the obesity-induced insulin resistance [88]. The negative correlation between HDLC level and obesity is well accepted [89].

#### Immunological pathways

PAK (*PAK2* and *PAK7*) genes feature in the high ranking T cell signaling pathway in both SP2 and SiMES datasets. *PRKC* (including *PRKCA* and *PRKCB*), along with *VAV* (*VAV2* and *VAV3*) genes also feature in various high ranking immunological pathways including T cell signaling, Pathogenic Escherichia Coli Infection and Natural Killer Cell Mediated Cytotoxicity. Genes from all 3 of these families are frequently top ranked in these pathways.

*PAK* and *VAV* are activated by antigens, and regulate the T cell cytoskeleton, indicating a possible impact on T cell shape and mobility. In a candidate gene association analysis, *PRKCA* was reported to be associated with HDLC at a nominally significant level, but was not significant after adjusting for multiple testing [90].

In summary, genes enriched in the above gene clusters and pathways may be relevant to heart muscle cell signal transduction, shape and migration, and may thus have functional relevance to the onset of cardiovascular diseases. Many highly ranked genes in our study are also involved in neurological pathways. For example polymorphisms in *CACNA1C* have been associated with bipolar disorder, schizophrenia and major depression [91]–[93]. This points to an interesting hypothesis that serum HDLC levels might be regulated not only by metabolism but also by neurological pathways, although the elucidation of any putative biological mechanism underlying such an association obviously exceeds the scope of this study.

Despite the well established links between lipid metabolism and PPAR signaling noted above, no genes in this high-ranked pathway fall in the top 30 gene rankings for either dataset (see Tables 7, 8 and 10). This could be because the association signal in this pathway is more widely distributed, compared to other high ranking pathways, perhaps indicating heterogeneity in genetic causal factors within our sample, so that different genes and SNPs are highlighted in different subsamples. This would result in reduced gene selection frequencies. Also, genes that overlap multiple putative causal pathways are more likely to be selected in a given subsample, meaning that associated genes mapping to pathways with relatively few overlaps may have lower selection frequencies. This may be the case with genes in the PPAR signaling pathway, whose 63 genes map to an average 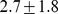 pathways. As a comparison, the 84 genes in the top-ranked dilated cardiomyopathy pathway map to an average 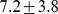 pathways.

Our study failed to highlight genes mapping to HDLC-associated SNPs identified in previous GWAS (see for example www.genome.gov/gwastudies for an up to date list). A primary reason for this is that the large majority of SNPs identified in previous studies do not map to pathways in our study, either because they fall in intergenic regions, or because they do not feature on the Illumina arrays used here. In addition our method is designed to highlight distributed, small genetic effects that accumulate across gene pathways, and so may fail to identify those SNPs and genes with significant marginal effects targeted by GWAS. Furthermore, where there are common mechanisms affecting phenotypes in both cohorts, we would expect to observe the most concordance between the two studies at the pathway level, followed by genes, and lastly SNPs. Indeed this increased heterogeneity at the SNP, and to a lesser extent at the gene level is one motivation for adopting a pathways approach in the first place [40], [58], [94]. This reduced concordance at the SNP level may be due to increased heterogeneity of genetic risk factors between the two datasets.

Some insight into these matters is gained by comparing our gene ranking results with those from a separate HDLC SNP GWAS in both SP2 and SiMES cohorts. By considering only SNPs that map to pathways in each cohort, we find that highly ranked genes using our method are significantly enriched amongst genes mapping to highly ranked SNPs in their respective GWAS. No pathway-mapped SNPs achieve statistical significance in either GWAS after correcting for multiple testing. There is thus some evidence that our method is able to highlight SNPs or genes with moderate or small marginal effects that would otherwise be missed using standard approaches, although this of course will depend on their distribution across pathways. As noted in our study, there is little concordance amongst the highest ranking GWAS SNPs and genes in both cohorts.

As observed in our simulation study using real genotype data, the tendency of the within-pathway lasso penalty to select one of a group of highly correlated SNPs at random can lead to reduced SNP selection frequencies within LD blocks harbouring causal SNPs. For this reason we do not report SNP rankings here. An alternative approach would be to consider a different penalty within selected pathways, for example the elastic net [13], which selects groups of correlated variables jointly, although this comes at the cost of introducing a further regularisation parameter to be tuned.

Finally, as with all pathways analyses, a number of limitations with this general approach should be noted. Despite great efforts, pathway assembly is still in its infancy, and the relative sparsity of gene-pathway annotations reflects the fact that our understanding of how the majority of genes functionally interact is at an early stage. As a consequence annotations from different pathways databases often vary [59], so that the choice of pathways database will impact results [58], [95]. Results are also subject to bias resulting from SNP to gene mapping strategies, so that for example SNP to gene mapping distances will affect the number of unmapped SNPs falling within gene ‘deserts’ [18]; SNPs may map to relatively large numbers of genes in gene rich areas of the genome; and the mapping of a SNP to its closest gene may obscure a true functional relationships with a more distant gene [39]. Indeed recent research from the ENCODE project indicates that functional elements may in fact be densely distributed throughout the genome [96], [97], and this information has the potential to radically alter future pathways analysis. These issues, together with the fact that pathways genetic association study methods are by construction designed to highlight distributed, moderate to small SNP effects, serve to further illustrate the point that pathways analysis should be seen as complementary to studies searching for single markers [6].

## Supporting Information

Information S1 Supplementary information and references.
(PDF)

Table S1 SP2 extended pathway ranks.
(TXT)

Table S2 SP2 extended gene ranks.
(TXT)

Table S3 SiMES extended pathway ranks.
(TXT)

Table S4 SiMES extended gene ranks.
(TXT)
