# Supplementary material for: Pathways-Driven Sparse Regression Identifies Pathways and Genes Associated with High-Density Lipoprotein Cholesterol in Two Asian Cohorts
Source: PLoS Genet. 2013 Nov 21;9(11):e1003939. doi: 10.1371/journal.pgen.1003939 (PMC3836716; doi:10.1371/journal.pgen.1003939)
Supplement: Information S1 — Supplementary information and references. (PDF) [file pgen.1003939.s001.pdf]

Supplementary Information

S1 SGL estimation algorithm

For the estimation of $\boldsymbol{\beta}^{SGL}$ we proceed by noting that the optimisation (1) is convex, and (in the case of non-overlapping groups) that the penalty is block-separable, so that we can obtain a solution using block, or group-wise coordinate descent (Tseng and Yun, 2009). For a single group, l, the corresponding minimising function is given by

$$f(\boldsymbol{\beta}_l) = \frac{1}{2}||\mathbf{y} - \mathbf{X}\boldsymbol{\beta}||_2^2 + (1-\alpha)\lambda w_l ||\boldsymbol{\beta}_l||_2 + \alpha\lambda||\boldsymbol{\beta}_l||_1. \tag{S.1}$$

An optimal solution for SNP coefficient β_j is then derived from the subgradient equations

$$-\mathbf{x}_j'(\hat{\mathbf{r}}_l - \sum_{k \neq j} \mathbf{x}_k \hat{\beta}_k - \mathbf{x}_j\beta_j) + (1-\alpha)\lambda w_l s_j + \alpha\lambda t_j = 0 \quad j = l_1, \ldots, l_{P_l}, \tag{S.2}$$

where $\hat{\beta}_k, k \neq j$ are the current estimates for other SNP coefficients in group l, and the group partial residual, $\hat{\mathbf{r}}_l = \mathbf{y} - \sum_{m \neq l} \mathbf{X}_m \hat{\boldsymbol{\beta}}_m$. Here s_j and t_j are the respective subgradients of $||\boldsymbol{\beta}_l||_2$ and $|\beta_j|$, with

$$s_j = \begin{cases} \frac{\beta_j}{||\boldsymbol{\beta}_l||_2} & \text{if } ||\boldsymbol{\beta}_l||_2 \neq \mathbf{0} \\ \in [-1,1] & \text{if } ||\boldsymbol{\beta}_l||_2 = \mathbf{0} \end{cases}$$
$$t_j = \begin{cases} \text{sign}(\beta_j) & \text{if } \beta_j \neq 0 \\ \in [-1,1] & \text{if } \beta_j = 0. \end{cases} \tag{S.3}$$

If $\boldsymbol{\beta}_l = \mathbf{0}$, that is group l is not selected by the model, then from (S.2)

$$-\mathbf{x}_j'\hat{\mathbf{r}}_l + (1-\alpha)\lambda w_l s_j + \alpha\lambda t_j = 0, \quad j = l_1, \ldots, l_{P_l}. \tag{S.4}$$

Substituting $\mathbf{a} = \mathbf{X}_l'\hat{\mathbf{r}}_l$ gives

$$a_j = (1-\alpha)\lambda w_l s_j + \alpha\lambda t_j, \quad j = l_1, \ldots, l_{P_l}$$

so that

$$s_j^2 = \frac{1}{(1-\alpha)^2\lambda^2 w_l^2}(a_j - \alpha\lambda t_j)^2, \quad j = l_1, \ldots, l_{P_l},$$

and

$$\sum_j s_j^2 = \frac{1}{(1-\alpha)^2\lambda^2 w_l^2}\sum_j (a_j - \alpha\lambda t_j)^2.$$

From (S.3), when $\boldsymbol{\beta}_l = \mathbf{0}$, $||s||_2 = (\sum_j s_j^2)^{\frac{1}{2}} \leq 1$, so that

$$\sum_j (a_j - \alpha\lambda t_j)^2 \leq (1-\alpha)^2 \lambda^2 w_l^2. \tag{S.5}$$

Also from (S.3), one further condition when $\boldsymbol{\beta}_l = \mathbf{0}$ is that $t_j \in [-1, 1]$. The values, $\hat{t}_j$ that minimise the left hand size of (S.5) are therefore given by

$$\hat{t}_j = \begin{cases} \frac{a_j}{\alpha\lambda} & \text{if } |\frac{a_j}{\alpha\lambda}| \leq 1 \\ \text{sign}(\frac{a_j}{\alpha\lambda}) & \text{if } |\frac{a_j}{\alpha\lambda}| > 1. \end{cases}$$

Substituting for a_j, we can then write the values for $a_j - \alpha\lambda t_j$ that minimise the left hand side of (S.5) as

$$\begin{aligned} a_j - \alpha\lambda t_j &= \begin{cases} 0 & \text{if } |\mathbf{x}_j'\hat{\mathbf{r}}_l| \leq \alpha\lambda \\ \text{sign}(\mathbf{x}_j'\hat{\mathbf{r}}_l)(|\mathbf{x}_j'\hat{\mathbf{r}}_l| - \alpha\lambda) & \text{if } |\mathbf{x}_j'\hat{\mathbf{r}}_l| > \alpha\lambda \end{cases} \\ &= S(\mathbf{x}_j'\hat{\mathbf{r}}_l, \alpha\lambda) \end{aligned}$$

for $j = l_1, \ldots, l_{P_l}$, where

$$S(\mathbf{x}_j'\hat{\mathbf{r}}_l, \alpha\lambda) = \text{sign}(\mathbf{x}_j'\hat{\mathbf{r}}_l)(|\mathbf{x}_j'\hat{\mathbf{r}}_l| - \alpha\lambda)_+ \tag{S.6}$$

is the lasso soft thresholding operator. Finally, we can now rewrite the condition for $\hat{\boldsymbol{\beta}}_l = \mathbf{0}$, (S.5) as

$$||S(\mathbf{X}_l'\hat{\mathbf{r}}_l, \alpha\lambda)||_2 \leq (1-\alpha)\lambda w_l, \tag{S.7}$$

Where the vector $S(\mathbf{X}_l'\hat{\mathbf{r}}_l, \alpha\lambda) = [S(\mathbf{x}_{l_1}'\hat{\mathbf{r}}_l, \alpha\lambda), \ldots, S(\mathbf{x}_{l_P}'\hat{\mathbf{r}}_l, \alpha\lambda)]$. Note that with $\alpha = 0$, this reduces to the group lasso group selection criterion.

In the case that $\boldsymbol{\beta}_l \neq \mathbf{0}$, that is group l is selected by the model, from (S.2) and (S.3) we see that $\beta_j = 0$ when

$$-\mathbf{x}_j'(\hat{\mathbf{r}}_l - \sum_{k \neq j} \mathbf{x}_k \hat{\beta}_k) \leq |\alpha\lambda|. \tag{S.8}$$

For completeness, we rewrite the criterion for selecting pathway l from (S.7) as

$$||S(\mathbf{X}_l'\hat{\mathbf{r}}_l, \alpha\lambda)||_2 > (1-\alpha)\lambda w_l \tag{S.9}$$

and the criterion for selecting SNP j in selected pathway l from (S.8) as

$$|\mathbf{x}_j'\hat{\mathbf{r}}_{l,j}| > \alpha\lambda \tag{S.10}$$

where $\hat{\mathbf{r}}_{l,j} = \hat{\mathbf{r}}_l - \sum_{k \neq j} \mathbf{x}_k \hat{\beta}_k$ is the SNP partial residual, obtained by regressing out the current estimated effects of all other predictors in the model, except for predictor j.

A number of methods for the estimation of $\boldsymbol{\beta}_l$ in the case that $||\boldsymbol{\beta}_l||_2 \neq \mathbf{0}$ have been proposed (Friedman, Hastie, and Tibshirani, 2010; Foygel and Drton, 2010; Liu and Ye, 2010; Simon et al., 2012). A complicating factor is the discontinuities in the first (and second) derivatives of s_j at $||\boldsymbol{\beta}_l||_2 = 0$, that is where $||\boldsymbol{\beta}_l||_2$ first moves away from zero, and of t_j when $\beta_j = 0$. As with GL, Friedman, Hastie, and Tibshirani (2010) describe a numerical method using coordinate descent, by combining a golden search over β_j with parabolic interpolation.

However we find this too computationally intensive for the large datasets we wish to analyse. Simon et al. (2012) propose an accelerated, block gradient descent method in which $\boldsymbol{\beta}_l$ is iteratively updated in a single step along the line of steepest descent of the block objective function until convergence. We instead use a block, coordinate-wise gradient descent (BCGD) method that uses a Newton update, similar to that proposed by Zhou et al. (2010), and we describe this below.

To update β_j from its current estimate, $\hat{\beta}_j$, we note from (S.2) and (S.3) that if $\hat{\beta}_j \neq 0$, the subgradient equation for predictor j is given by

$$\partial_j = -\mathbf{x}_j'(\hat{\mathbf{r}}_l - \mathbf{X}_l\hat{\boldsymbol{\beta}}_l) + (1-\alpha)\lambda w_l \frac{\hat{\beta}_j}{||\hat{\boldsymbol{\beta}}_l||_2} + \alpha\lambda \cdot \text{sign}(\hat{\beta}_j). \tag{S.11}$$

We then descend along the gradient at $\hat{\beta}_j$ towards the minimum using Newton's method. The Newton update, β_j^*, is then given by

$$\begin{aligned} \hat{\beta}_j^* &= \hat{\beta}_j - \frac{\partial_j}{\partial_j'}, \\ \text{where} \quad \partial_j' &= 1 + \frac{(1-\alpha)\lambda w_l}{||\hat{\boldsymbol{\beta}}_l||_2}\left(1 - \frac{\hat{\beta}_j^2}{||\hat{\boldsymbol{\beta}}_l||_2^2}\right) \end{aligned} \tag{S.12}$$

is the derivative of (S.11) at $\hat{\beta}_j$. The update (S.12) is repeated until convergence.

We must also deal with the case where $\hat{\beta}_j = 0$. Here we adopt a slightly different strategy, since the partial derivative, t_j of β_j is not continuous. We avoid this discontinuity by testing the 'directional derivatives', ∂_j^+ and ∂_j^-, respectively representing the partial derivatives at $\beta_j = 0$ in the direction of increasing and decreasing β_j. Recalling that we are dealing with the case $||\boldsymbol{\beta}_l||_2 \neq \mathbf{0}$, at $\beta_j = 0$ the group penalty term in (S.11) disappears. That is, once a group is selected by model it becomes easier for each SNP coefficient to move away from zero. The two directional derivatives are then given by

$$\begin{aligned} \partial_j^+ &= -\mathbf{x}_j'(\hat{\mathbf{r}}_l - \mathbf{X}_l\hat{\boldsymbol{\beta}}_l) + \alpha\lambda \\ \partial_j^- &= -\mathbf{x}_j'(\hat{\mathbf{r}}_l - \mathbf{X}_l\hat{\boldsymbol{\beta}}_l) - \alpha\lambda. \end{aligned} \tag{S.13}$$

Since the minimising function (S.1) is convex, there are three possible outcomes, and we substitute for ∂_j in (S.12) accordingly:

$$\partial_j \leftarrow \begin{cases} \partial_j^- & \text{if} \quad \partial_j^- > 0 \quad \text{and} \quad \partial_j^+ > 0 \\ \partial_j^+ & \text{if} \quad \partial_j^- < 0 \quad \text{and} \quad \partial_j^+ < 0 \\ 0 & \text{if} \quad \partial_j^- > 0 \quad \text{and} \quad \partial_j^+ < 0 \end{cases} \tag{S.14}$$

In the third case, $f(\boldsymbol{\beta}_l)$ is increasing either side of $\beta_j = 0$, so that $\hat{\beta}_j$ must remain at zero. We can then proceed with the standard Newton update (S.12).

Finally, since the Newton update may occasionally overstep the minimum (where $\partial_j = 0$), a simple remedy proposed by Zhou et al. (2010) is to check that $f(\boldsymbol{\beta}_l)$ is decreasing at each iteration. If this is not the case, then the step size in (S.12) is halved. The complete algorithm for SGL estimation using BCGD is presented in Box 1.

One remaining practical issue is the obtaining of a value for λ_{max}, the smallest value of λ at which no groups are selected by the model. Noting that $\hat{\mathbf{r}}_l = \mathbf{y}$ when no groups are selected, from (S.7) we obtain the smallest value, λ_l^{min}, for the minimum value of λ at which group l is not selected as

$$\lambda_l^{min} = \frac{||S(\mathbf{X}_l'\mathbf{y}, \alpha\lambda_l^{min})||_2}{(1-\alpha)w_l} \tag{S.15}$$

We can solve this in its quadratic form by first setting an upper bound for λ at the point λ_l^*, where the soft thresholding function $S(\mathbf{X}_l'\mathbf{y}_l, \alpha\lambda) = \mathbf{0}$, that is when no SNPs are selected by the model. We then obtain the solution by solving

$$||S(\mathbf{X}_l'\mathbf{y}, \alpha\lambda_l^{min})||_2^2 - (1-\alpha)^2(\lambda_l^{min})^2 w_l^2 = 0 \quad 0 < \lambda_l^{min} < \lambda_l^* \tag{S.16}$$

for λ_l^{min}, where

$$\lambda_l^* = \max_j \frac{|\mathbf{x}_j'\mathbf{y}|}{\alpha}, \quad j = l_1, \ldots, l_{P_l}.$$

We do this using the 1d root-finding function, *brentq*, in Python's *scipy* library. Finally, we obtain a value for λ_{max} as

$$\lambda_{max} = \max_l \lambda_l^{min}, \quad l = 1, \ldots, L. \tag{S.17}$$

S2 SGL with overlaps

We assume that $\mathbf{X}$ and $\boldsymbol{\beta}$ have been expanded to account for overlaps, but we drop the * notation for clarity. We proceed as before by solving the block-separable optimisation (4) for each group or pathway in turn. However, for overlapping pathways, the assumption of pathway independence requires that each $\mathbf{X}_l, (l = 1, \ldots, L)$ is regressed against the full phenotype vector $\mathbf{y}$ rather than the partial residual, $\hat{\mathbf{r}}_l$. With this in mind, the revised subgradient equations for group l (S.2) are given by

$$-\mathbf{x}_j'(\mathbf{y} - \sum_{k\neq j}\mathbf{x}_k\hat{\beta}_k - \mathbf{x}_j\beta_j) + (1-\alpha)\lambda w_l s_j + \alpha\lambda t_j = 0 \quad j = l_1, \ldots, l_{P_l}. \tag{S.18}$$

The estimation for group l then proceeds as described in the previous section, but with the partial residual $\hat{\mathbf{r}}_l$ replaced by $\mathbf{y}$, so that the group sparsity condition (S.7) for $||\hat{\boldsymbol{\beta}}_l||_2 = \mathbf{0}$ becomes

$$||S(\mathbf{X}_l'\mathbf{y}, \alpha\lambda)||_2 \leq (1-\alpha)\lambda w_l. \tag{S.19}$$

As before, where group l is selected by the model, the update for β_j, with current estimate $\hat{\beta}_j$, is derived from the partial derivative (S.11), which under the independence assumption is given by

$$\partial_j = -\mathbf{x}_j'(\mathbf{y} - \mathbf{X}_l\hat{\boldsymbol{\beta}}_l) + (1-\alpha)\lambda w_l \frac{\hat{\beta}_j}{||\hat{\boldsymbol{\beta}}_l||_2} + \alpha\lambda \cdot \text{sign}(\hat{\beta}_j), \tag{S.20}$$

for $j = l_1, \ldots, l_{P_l}$. The Newton update (S.12) remains the same. When $\hat{\beta}_j = 0$, the revised directional derivatives (S.13) are given by

$$\begin{aligned} \partial_j^+ &= -\mathbf{x}_j'(\mathbf{y} - \mathbf{X}_l\hat{\boldsymbol{\beta}}_l) + \alpha\lambda \\ \partial_j^- &= -\mathbf{x}_j'(\mathbf{y} - \mathbf{X}_l\hat{\boldsymbol{\beta}}_l) - \alpha\lambda. \end{aligned} \tag{S.21}$$

As before the conditions for SNP sparsity within a selected group are determined by (S.14).

The value of λ_{max}, the smallest λ value at which no group is selected by the model, is determined in the same way as before, since this procedure (described in (S.15), (S.16) and (S.17)) does not depend on $\hat{\mathbf{r}}_l$.

Importantly, since each group is regressed independently against the phenotype vector $\mathbf{y}$, there is no block coordinate descent stage in the estimation, that is the revised algorithm utilises only coordinate gradient descent within each selected pathway. For this reason we use the acronym SGL-CGD for the revised algorithm. The new algorithm is described in Box 2. Note that since the block coordinate descent stage is avoided, the new algorithm has the added benefit of being much faster than would otherwise be the case.

S3 Simulation study 1

A baseline phenotype, y is sampled from $\mathcal{N}(10, 1)$. To generate SNP effects, we first select a single pathway, $\mathcal{G}_l$, at random. From this pathway we randomly select 5 SNPs to from the set $\mathcal{S} \subset \mathcal{G}_l$ of causal SNPs. At each MC simulation we generate a genetic effect and adjust y so that

$$y^* = y + w$$

where

$$w = \delta \sum_{k \in \mathcal{S}} \zeta_k x_k.$$

Here δ controls the overall additive genetic effect on phenotype y due to all casual SNPs in $\mathcal{S}$, and ζ_k determines the contribution from causal SNP k, with $\sum_{k \in \mathcal{S}} \zeta_k = 1$. In our simulations we maintain a constant overall genetic effect size,

$$\gamma = \mathrm{E}(w)/\mathrm{E}(y)$$

across all affected phenotypes, so that γ represents the proportionate increase in the mean value of y due to all genetic effects. We also set $\zeta_k = 1/5$, for $k \in \mathcal{S}$, so that the contribution from each causal SNP allele is equal. This enables us to determine δ for a given γ as

$$\delta = \frac{5\gamma \mathrm{E}(y)}{2 \sum_{k \in \mathcal{S}} m_k}.$$

Note that for constant γ, the proportionate effect on the mean value of y due to SNP k is MAF dependent, and is given by $2\delta m_k/\mathrm{E}(y)$.

S4 Weight tuning for bias reduction

For fixed α, and with λ tuned to select a single pathway, we need to establish which pathway enters the model first, as λ is reduced from its maximal value, λ_{max}. From (S.17), at phenotype permutation r, the pathway $\hat{\mathcal{C}}_r$ selected with permuted phenotype $\mathbf{y}_r$ is given by

$$\hat{\mathcal{C}}_r = \arg\max_l \lambda_l^{min}, \quad , l = 1, \ldots, L.$$

λ_l^{min} is obtained by solving

$$\lambda_l^{min} = \frac{||S(\mathbf{X}_l'\mathbf{y}_r, \alpha\lambda_l^{min})||_2}{(1-\alpha)w_l},$$

using the procedure described at the end of Section S1. For R permutations of the phenotype vector, $\mathbf{y}$, the empirical pathway selection frequency distribution is then given by

$$\Pi^*(\mathbf{w}) = \frac{1}{R}\sum_{r=1}^{R}\{\hat{\mathcal{C}}_r = l\}, \quad l = 1, \ldots, L.$$

S5 Investigation of effect of pathway size on pathway selection

We extend the simulation framework described for Simulation Study 1 by allowing pathway size, measured by number of SNPs, to vary. Specifically, we follow the same scenario for generating genotypes, but generate 20 non-overlapping pathways varying in size from 10 to 200 SNPs, in increments of 10. Phenotypic effects are generated as previously described, after randomly selecting 5 causal SNPs from a single randomly selected pathway at each MC simulation. We use an 'intermediate' effect size, $\gamma = 0.05$ (see Figure 3 in the main text), to best assess any potential variation in pathway selection power, defined as the proportion of simulations where the causal pathway is correctly selected. We perform 10,000 MC simulations, with λ tuned to ensure a single pathway is selected at each simulation, and with $\alpha = 0.85$. We use a uniform pathway weight vector, $\boldsymbol{w} = 1$. Pathway selection power is plotted against pathway size in Figure S1. There is no significant relationship between the two (linear regression: slope = 0.00013; $r^2 = 0.12$; $p = 0.13$).

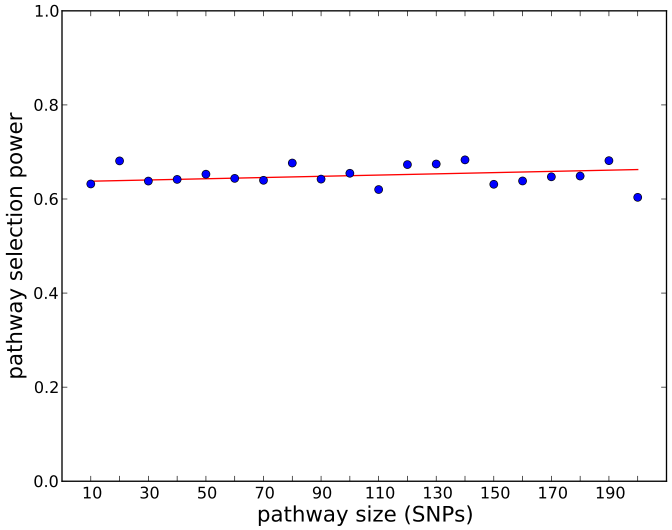

Figure S1: Variation of power to select a single causal pathway with causal pathway size.

S6 Comparisons with HDLC SNP GWAS

Here we present some further details on our method for comparing SGL gene rankings with those obtained from separate HDLC SNP GWAS studies for SP2 and SiMES cohorts. GWAS

results form part of an ongoing multi-cohort GWAS study, and so cannot be reported in detail.

Our comparison method works as follows:

1. Using only SNPs that map to pathways in our study, we ranked SNP GWAS results for each cohort by ascending p-value.
2. We next obtained a corresponding gene ranking by ranking genes according to the most significant mapped SNP. This gives us 'GWAS rankings' for all the genes in our study (4,734 in the SP2 cohort, and 4,751 in SiMES).
3. Looking only at the top 50 genes ranked by our method in each cohort, we obtained a mean ranking for each of these genes in their respective SNP GWAS.
4. We then tested the null hypothesis that the top 50 genes ranked by our method are not significantly enriched amongst highly ranked genes in their respective GWAS using the following permutation test for each GWAS:
   (a) pick 50 genes at random from the complete list of genes ranked in the GWAS
   (b) obtain a permutation ranking score as the mean GWAS ranking achieved by all 50 randomly selected genes
   (c) compute 1,000,000 such scores, each with a new random selection of 50 genes
   (d) finally, compute a permutation p-value as the proportion of permutations where the permutation mean ranking score is less than or equal to the empirical mean ranking score.

For both cohorts we obtain $p < 10^{-6}$, indicating that genes highly-ranked by our method are significantly enriched amongst highly ranked genes in each respective GWAS.

References

Foygel, Rina and Mathias Drton (2010). "Exact block-wise optimization in group lasso and sparse group lasso for linear regression".

Friedman, Jerome, Trevor Hastie, and Robert Tibshirani (2010). "A note on the group lasso and a sparse group lasso", pp. 1–8. arXiv:/arxiv.org/abs/1001.0736 [http:].

Liu, Jun and Jieping Ye (2010). "Fast Overlapping Group Lasso", pp. 1–14. arXiv:1009.0306.

Simon, Noah et al. (2012). "A sparse-group lasso". *Journal of Computational and Graphical Statistics* In press, pp. 1–13.

Tseng, Paul and Sangwoon Yun (2009). "A coordinate gradient descent method for nonsmooth separable minimization". *Mathematical Programming* 117.1, pp. 387–423.

Zhou, Hua et al. (2010). "Association Screening of Common and Rare Genetic Variants by Penalized Regression." *Bioinformatics (Oxford, England)* 26.19, pp. 2375–2382.
